# Interpretable machine learning for dementia: A systematic review

**DOI:** 10.1002/alz.12948

**Published:** 2023-02-03

**Authors:** Sophie A. Martin, Florence J. Townend, Frederik Barkhof, James H. Cole

**Affiliations:** ^1^ Centre for Medical Image Computing Department of Computer Science University College London London UK; ^2^ Dementia Research Centre Queen Square Institute of Neurology University College London London UK; ^3^ Amsterdam UMC, Department of Radiology & Nuclear Medicine Vrije Universiteit Amsterdam Netherlands

**Keywords:** dementia, diagnosis, explainable artificial intelligence, interpretability, machine learning, mild cognitive impairment

## Abstract

**Introduction:**

Machine learning research into automated dementia diagnosis is becoming increasingly popular but so far has had limited clinical impact. A key challenge is building robust and generalizable models that generate decisions that can be reliably explained. Some models are designed to be inherently “interpretable,” whereas post hoc “explainability” methods can be used for other models.

**Methods:**

Here we sought to summarize the state‐of‐the‐art of interpretable machine learning for dementia.

**Results:**

We identified 92 studies using PubMed, Web of Science, and Scopus. Studies demonstrate promising classification performance but vary in their validation procedures and reporting standards and rely heavily on popular data sets.

**Discussion:**

Future work should incorporate clinicians to validate explanation methods and make conclusive inferences about dementia‐related disease pathology. Critically analyzing model explanations also requires an understanding of the interpretability methods itself. Patient‐specific explanations are also required to demonstrate the benefit of interpretable machine learning in clinical practice.

## 1 INTRODUCTION

Traditional dementia diagnosis typically relies on longitudinal clinical observations, medical history, and symptoms of cognitive decline such as impaired memory and visuospatial deficits, often supported by imaging findings. Computer‐aided decision tools are increasingly making use of machine learning to speed up diagnosis, provide support where expert knowledge is sparce, and reduce subjectivity.^1^ Machine learning models have been shown to perform as well as, or even exceed the accuracy of predictions made from imaging by radiologists, as they can exploit the rich information present in dense, high‐dimensional data.^2^ They also show promise at identifying those at risk earlier in the disease trajectory, because relying on longitudinal clinical observations usually means that the disease has already progressed beyond the point that preventive protocols or adjustments can be effective. However, despite promising results in medical research, computer‐aided tools have yet to be widely adopted in the clinic. A major factor in this is the black‐box nature of predictive models, which makes them difficult to interpret and, ultimately, to trust.^3^, ^4^

**FIGURE 1 alz12948-fig-0001:**
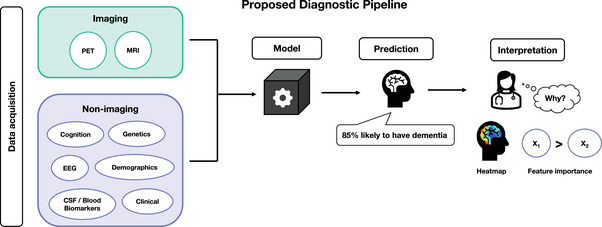
We propose a diagnostic pipeline that starts with data acquisition through to clinical interpretation. Data can be categorized into imaging and non‐imaging groups. Data items can be used individually or combined to make a prediction. A model can be trained to predict the probability an individual's likelihood to have or develop dementia using these data. A clinician using this model may wish to interpret the result, to understand “why” this person has been classified as having dementia, which could influence the most appropriate treatment response or help to confirm their own diagnosis. The interpretation method depends on the model and data types involved. Most methods either produce heatmaps, which visualize influential regions or use techniques to rank the most important features

Interpretable machine learning (IML) often used synonymously with explainable artificial intelligence (XAI), can be used to explain the output of predictive models by (1) describing the mechanism by which the model generates its decision, (2) highlighting which of the input features are most influential on the decision, or (3) producing examples that maximize its confidence for a specific outcome. As shown in Figure 1, an interpretation stage can be introduced into the machine learning pipeline that can confirm a clinician's diagnosis or provide patient‐specific evidence of the disease. Although arguments for explainability often focus on trust,^5^, ^6^, ^7^, ^8^, ^9^, ^10^, ^11^, ^12^ other goals include fairness, accessibility, interactivity, and exploration; the process of model interpretation may uncover new knowledge about the model, data, or underlying disease.^13^ There is also growing pressure from a legal standpoint to provide explanations—both to the clinician and the patient. This became evident when new European General Data Protection Regulations (GDPRs) were introduced in 2018 calling for more transparency, and individuals were given a “right to explanation.”^14^ Moreover, a report from the National Health Service (NHS) Artificial Intelligence (AI) Lab and Health Education England published in May 2022 noted that “adopting AI technologies [is] at a critical juncture” with calls for “appropriate confidence” in AI for both health care workers and the public. The phrase “appropriate confidence” shifts focus way from trust (a subjective and qualitative measure) to reflect how users must be able to “make context‐dependent value judgments and continuously ascertain the appropriate level of confidence in AI‐derived information.”^15^ This distinction mirrors the difference between the use of AI for lone decision‐making versus as a decision‐support tool, with the latter being the focus of translational research.

The field of IML has grown rapidly over the last 20 years,^3^, ^4^, ^13^ particularly in tasks involving natural language processing or computer vision. This rapid growth has led to inconsistencies in the terminology used to describe such methods, making it difficult to identify relevant studies. Although many reviews on IML introduce taxonomies that bring clarity to the different methods,^16^ there is still inconsistency across research papers when incorporating explanation methods in their analysis. In dementia studies specifically, coupled with the variety of data available for differential diagnosis and prognosis, this has led to a complex landscape of methods that makes it hard to identify best practice. There is also variability across machine learning studies in the reporting of implementation details, which can also inhibit translation to clinical practice. This systematic review aims to summarize current progress and highlight areas for improvement to allow dementia researchers to better navigate this emerging field.

## 2 BACKGROUND

The landscape of interpretable machine learning has grown rapidly with the development of new techniques and their applications across domains. Details on these methods and their properties can be found in resources such as Christoph Molnar's guide.^17^ Recent reviews of interpretable machine learning have introduced frameworks (taxonomies) that summarize their properties, provide a visual aid, and promote consistency across future work.^13^, ^16^, ^18^

**RESEARCH IN CONTEXT**

1. **Systematic Review**: We reviewed the literature and identified 92 studies published by March 1, 2022 (PubMed, Scopus, Web of Science) that use machine learning to predict dementia and provide evidence of explaining the predictions.
2. **Interpretation**: Studies demonstrated promising classification performance, with many incorporating neuroimaging into their models and using methods such as class activation mapping and occlusion to explain the models predictions. Our findings align with existing analyses of machine learning applications for dementia including an over‐reliance on large open‐source datasets, inconsistent reporting of sample sizes, and insufficient assessments of model generalisability.
3. **Future Directions**: Future work should incorporate clinicians into the validation of model explanations to assess their clinical utility and explore the impact of model explanations on trust. There are also opportunities to explore inherently interpretable models that produce pixel‐level explanations and develop context‐specific measures of robustness.

### 2.1 Properties of interpretable methods

Here we introduce some of the key properties of model interpretation methods. Understanding their properties can help researchers to critically analyze the resulting explanations, and identify which methods are most appropriate for a given clinical scenario or question. These properties include whether they are intrinsic or post hoc, model‐agnostic or model‐specific, and whether they produce model, individual, or group level explanations. However, the categorization of these methods varies across the literature and some methods can fall into more than one group. Therefore, these properties and the methods associated with them are best considered within the context of the predictive task.

#### 2.1.1 Intrinsically interpretable models versus post hoc interpretation methods

Machine learning methods such as linear regression, k‐nearest neighbors, decision trees, and their extensions can be classified as intrinsically interpretable because for a given set of inputs and outputs, the end‐user can easily trace how the inputs have been used to arrive at the final probability, value, or prediction often via a formula or rule‐based framework. For example, linear regression predictions are a weighted sum of the input features, or subset of important features based on their assigned weights if regularization techniques (such as Least Absolute Shrinkage and Selection Operator (LASSO)) are used. Similarly, decision trees can be interpreted because their final probabilistic outputs or values are derived via a rule‐based framework allowing users to trace the decision boundaries from input to output.

**FIGURE 2:**
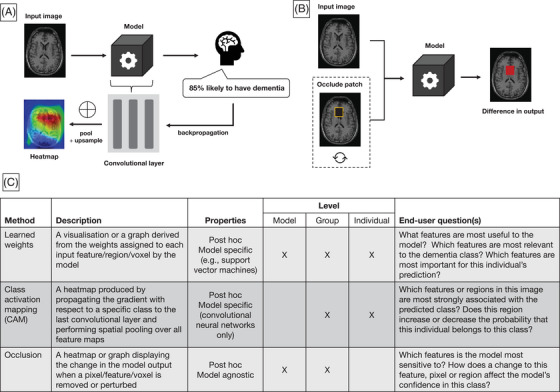
Example brain scan.^80^ (A) High‐level illustration of class activation mapping for image‐based classification. An individual case is fed through the model and the output probability is backpropagated to the last convolutional layer in the network. The values across all filters in the layer are pooled (typically global pooling is used) and up‐sampled to the input space to produce a heatmap that indicates which features have most influence on the final prediction. (B) Illustration of occlusion methods for interpreting image‐based models. Occlusion‐based maps are produced by comparing the output of the model between the original image and the image when a patch is removed (or perturbed, e.g., using a fixed value). This process is repeated for different patches to build up an image that indicates the model's sensitivity to a given patch. (C) Descriptions of the top three interpretation methods, their properties, and example questions of their use‐case in a clinical setting

Post hoc interpretation methods[Fig alz12948-fig-0001] involve an additional step of exploration after training, in which the trained model is probed or manipulated to generate information on how input features influence the output. Such methods include perturbation methods, backpropagation, feature relevance ranking, or example‐based explanations. Perturbation methods, sometimes referred to as sensitivity analysis, involve systematically changing the input data (e.g., removing features) and observing its effect on the output. This allows users to determine whether the model is more sensitive to specific features or regions. Backpropagation is often used for “black‐box” models such as deep neural networks, where the underlying predictive process is complex due to non‐linear operations and high‐dimensional input data. These methods utilize the weights learned during training to propagate the output probability back into the input space, resulting in heatmaps that highlight the importance of pixels, regions, or features. By probing the model after training, post hoc approaches have the advantage of deriving explanations without compromising accuracy for instances where deep models outperform less‐complex linear approaches. Figure 2 contains schematic representations of two post hoc methods: class activation mapping (CAM) and occlusion, their properties, and questions an end‐user could use to determine which method is most appropriate.

Creating intrinsically interpretable models is more challenging for neural networks due to their complex architectures. However, examples include ProtoPNet,^19^ a neural network for which the final classification is generated by chaining learned “prototypes” (or parts of the image) through a transparent algorithm. Transformer networks can also be considered as an interpretable deep learning models because the self‐attention mechanism that generates their output can also be used to highlight important regions or features.^20^, ^21^ Although transformers were designed initially for use in natural language processing tasks, the evolution of vision transformers has led to a rise in use across the medical imaging domain.^22^, ^23^ Transformers and their vision counterparts show promise for being able to maintain the predictive power of deep neural networks while incorporating attention into their architectures.

#### 2.1.2 Model‐agnostic versus model‐specific

Methods such as occlusion are model agnostic: they can be applied to any predictive model. Other examples include Shapley values,^24^ local interpretable model explanations (LIME),^12^ and counterfactual examples. Counterfactual examples explain models by producing synthetic representations of the input data that maximize the probability of a chosen outcome. For instance, warping the input image from a healthy participant so that the model believes it is likely to belong to a person from the dementia class would highlight features that the model associates with the disease. However, the generalizability of model‐agnostic methods is limited as they are often unable to produce fine‐grained explanations.

Model‐specific methods may be more appropriate for interpreting neural networks, where information is needed on a pixel or voxel level. Examples include Grad‐CAM^25^ and layer‐wise relevance propagation.^26^ However, model‐specific methods rely on assumptions about the underlying architecture such as the presence of convolutional layers to produce an explanation, which limits their use for comparing results across model types.

#### 2.1.3 Model‐ versus individual‐ versus group‐level explanations

IML methods can also be categorized according to the application‐level of the explanation. Model‐level (or global) explanations describe the overall model and can be used to identify the most important features across all classes. Methods such as LIME can be used to produce individual‐level explanations, which describe the important features for a specific case. This is likely to be more useful in clinical settings, as patient‐specific explanations can be used to inform future treatment or confirm a diagnosis. In many cases, a single IML method can be used to produce explanations across several levels. For example, group‐level explanations can be produced by combining or averaging the individual explanations produced by LIME for each subject group. On the other hand, perturbation methods such as occlusion are not useful for deriving patient‐specific explanations because they rely on the learned model's behavior across all examples seen during training. Moreover, some methods require the class of interest to be specified to calculate the explanation such as class activation mapping (or CAM) and layer‐wise relevance propagation (LRP). In these cases, the output is produced based on the gradient of the loss function with respect to a specific class (via backpropagation) and the result is a heatmap representing the relevance to that group. Many neural network–based approaches rely on backpropagation and differ mainly in the way non‐linear operations are handled and propagated.

### 2.2 Study motivation

Although there are several reviews that summarize IML literature across medical imaging and computer vision,^2^, ^3^, ^27^ few focus on their application to dementia research and machine learning. Borchert and colleagues recently reviewed neuroimaging‐based machine learning for dementia prediction, with recommendations on how to increase impact in memory clinic settings.^28^ Similarly, Thibeau‐Sutre and colleagues performed a review on interpretable methods in neuroimaging, where they highlighted various methods and assessed their reliability.^29^ However, to our knowledge this systematic review is the first to consider both imaging and non–imaging‐based machine learning methods for dementia diagnosis, where model interpretability is a specific inclusion criterion. Our review is also not limited to Alzheimer's disease but considers approaches that include a range of dementia‐causing neurodegenerative diseases. This review aims to (1) summarize the different approaches to interpretable or explainable dementia prediction, (2) report and highlight the variability in study design and how this impacts clinical interpretability, and (3) offer recommendations for dementia researchers that wish to incorporate interpretable methods in future work.

## 3 MATERIALS AND METHODS

We conducted a systematic review of studies that used machine learning or deep learning for diagnostic classification of dementia and interpret the results either using post hoc analysis or inferring from an interpretable model. A protocol for this systematic review was registered on PROSPERO (ID: CRD42021291992).^30^ PROSPERO is an international prospective register of systematic reviews that helps to avoid duplication and reduce reporting bias. A database search was used to identify reports published before March 1, 2022, across PubMed, Scopus, and Web of Science. We constructed our search query by linking four key concepts together: dementia, classification, machine learning, and interpretability. The search query run on each database is given below (adapted for each database) and all terms were searched across titles, abstracts, and keywords (if available):

(“dementia” OR “alzheimer*”)
**AND**
(“predict*” OR “classif*” OR “diagnosis”)
**AND**
(“deep learning” OR “machine learning” OR “neural network*”)
**AND**
(“explain*” OR “interpret*” OR “saliency” OR “Grad‐CAM” OR “Layer?wise relevance propagation” OR “occlusion” OR “visuali*” OR “transformer”)

This returned 219 records on PubMed, for which the MeSH terms “dementia” and “diagnosis, computer assisted” were also used. On Scopus the query returned 531 records and on Web of Science the query returned 308 records. A total of 530 records were removed with EndNote's automated de‐duplication tool and manual assessment before screening.

### 3.1 Screening process

All records were screened using a two‐stage process using two independent reviewers based on: (1) title and abstract only and (2) full‐text. The inclusion and exclusion criteria used to filter studies are summarized below:

1. **Article type**
  - Inclusion: Any published original research paper (or pre‐prints) in peer‐reviewed academic journals or conferences.
  - Exclusion: Conference proceedings, corrections, erratum's, reviews, and meta‐analyses.
2. **Task**
  - Inclusion: Application of machine learning to do one or both of the following: (i) classify dementia patients from healthy controls or mild cognitive impairment patients, (ii) classify individuals that convert from stable/early mild cognitive impairment to progressive/late mild cognitive impairment or dementia.
  - Exclusion: Unsupervised algorithms (e.g., clustering methods, generative adversarial networks) or applications of supervised machine learning to non‐diagnostic tasks (e.g., segmentation, brain atrophy, brain parcellation, brain‐age prediction, prediction of cognitive assessment scores, genome‐wide analysis, survival analysis).
3. **Application to dementia**
  - Inclusion: Studies with patient groups based on a clinical diagnosis of dementia, Alzheimer's disease, or phenotypic syndrome (e.g., frontotemporal lobar degeneration).
  - Exclusion: Studies with patient groups based on other neurodegenerative diseases (e.g., Huntington's or Parkinson's disease) without an accompanying dementia diagnosis.
  - Exclusion: Classification of other forms of neurodegeneration (e.g., multiple sclerosis, traumatic brain injury, stroke, or mild cognitive impairment only).
4. **Model interpretability**
  - Inclusion: Studies must refer to the interpretability of the classification model in the abstract or provide example model explanations in the main text.
  - Exclusion: Classical data‐driven feature selection or dimensionality reduction studies (e.g., principal component analysis).
5. **Data**
  - Inclusion: Studies must report experimental details including the type of prediction model used, and at least one of the following performance metrics: accuracy, area under the curve, precision, recall, sensitivity, or specificity within the text or figures.
6. **Not human**
  - Exclusion: Non‐human studies, for example, mouse models.

**FIGURE 3:**
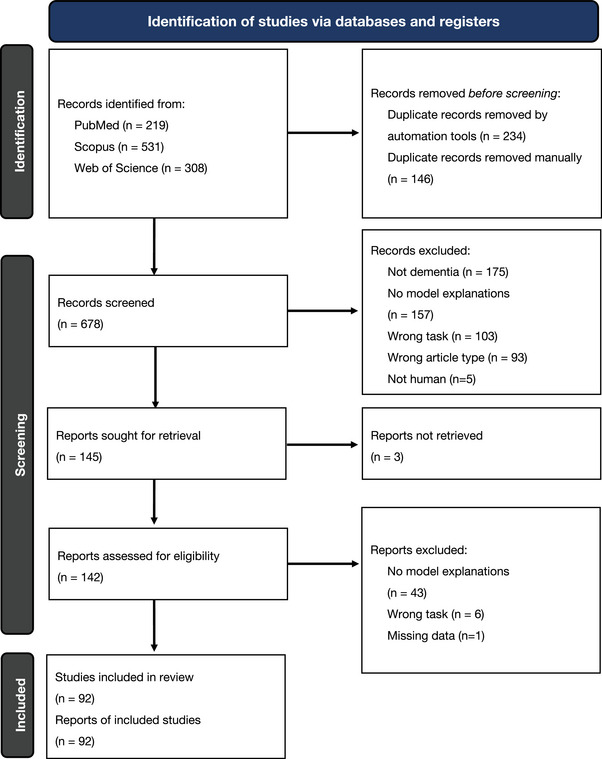
PRISMA flowchart outlining the screening strategy used to identify relevant studies.^31^ A search was performed on three databases: PubMed, Scopus, and Web of Science and returned a total of 1058 records. After duplication removal and screening against eligibility criteria, 92 full‐text studies were left for inclusion in this review. Studies were excluded for not being focused on dementia (non‐dementia), not clearly demonstrating interpretable methods or model explanations (no model explanations), focusing on the wrong task, for example, regression or survival analysis (wrong task), missing data (such as performance metrics), or being of the wrong article type (review papers, book chapters, conference proceedings)

A PRISMA flowchart^31^ describing the study selection process is shown in Figure 3. For title and abstract screening, any papers that were on the borderline for inclusion were assessed blindly by a second reviewer. Any studies without consensus automatically progressed onto the second screening stage. This led to 144 papers requiring a full‐text screening for inclusion. We removed 48 reports upon reading the full‐text for failing the eligibility criteria. We contacted the authors for any full‐text papers we could not retrieve online. We applied the same blind review process for borderline full‐text reports to obtain a final list of 92 publications. For these included studies we extracted the following information where applicable:

- Data sources
- Group labels / diagnostic categories
- Sample size (total number of participants across datasets)
- Validation or test split procedure used
- Whether quality control or data augmentation had been performed
- The type of input data used by the model
- The predictive model used
- Whether the task was binary or multiclass
- Performance metrics (e.g., accuracy, precision, recall, specificity, sensitivity)
- The interpretability method
- Important features (or regions) derived from the model
- Whether attempts had been made to validate the interpretability method
- Whether the code has been made publicly available

### 3.2 Risk of bias

During screening, 200 papers were excluded because they did not address model interpretability. However, ascertaining what qualifies as interpretable raises important questions about what counts as an “explanation.” For instance, many studies involved the use of feature selection methods or dimensionality reduction prior to model training, for example, using principal component analysis. These studies may refer to the reduced features as being “interpretable”; however, we do not include these studies here as the emphasis was on the input features and not the trained prediction models. It is also important to note that not all studies explicitly mention the interpretability of their model despite them being inherently interpretable. This is particularly relevant for earlier studies that use classical regression techniques but may not have been captured by our search query. In addition, disease progression models can be used to predict diagnosis and can be inherently interpretable.^32^ However, they are not included in our review, as these models typically rely on unsupervised clustering methods. As such there is a risk of bias, as we focus only on machine learning studies that explicitly mention interpretability and include example inferences.

## 4 RESULTS

**FIGURE 4:**
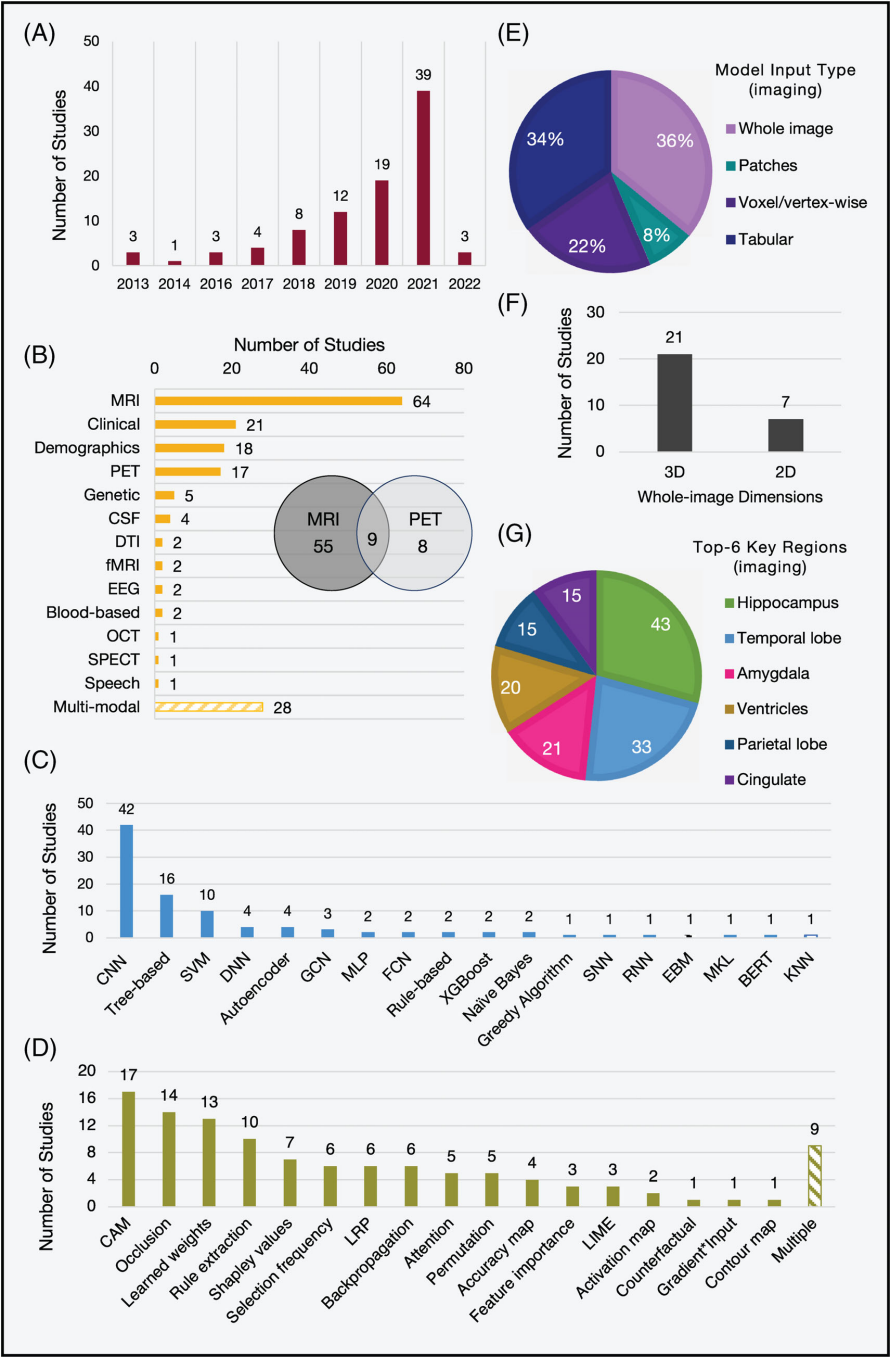
Key characteristics identified across all 92 included studies. (A) The number of papers per year. (B) The modalities used in the study. (C) The type of machine learning or deep learning model used to predict and interpret. (D) The type of interpretability method. For imaging studies only (*n* = 77): (E) The type of input data used by the predictive model. Tabular is used to denote measures such as cortical thickness or volume. One study used both tabular and voxel‐wise features. (F) The proportion of studies that used 3D or 2D whole images. (G) The top six important brain regions identified. Blood‐based: Blood‐based biomarkers; CAM, class activation mapping; [C/D/S/R]NN, [convolutional/deep/spiking/recurrent] neural network, tree‐based (e.g., random forests, decision trees); CSF, cerebrospinal fluid, DTI: diffusion tensor imaging; EBM: explainable boosting machine; EEG, electroencephalography; [F/G]CN, [fully/graph]convolutional network; [f]MRI: [functional] magnetic resonance imaging; KNN, k‐nearest neighbors; LIME, local interpretable model explanations; LRP, layer‐wise relevance propagation; MKL, multi‐kernel learning; OCT, optical coherence tomography; PET, position emission tomography; SPECT, single‐photon emission computed tomography; SPHOG: spatial pyramid histogram of oriented gradient.

We reviewed and extracted data from all included studies using Excel to highlight trends in the study design, performance, and IML methods used. The key findings are summarized in Figure 4 and extracted data items can be found in the Supplementary Material.

### 4.1 Study details

The key study details across all studies can be found in Table S1. Here we summarize the trends seen in the data sets used, variability in sample size, and use of neuroimaging data.

We identified that 67 of 92 studies used the Alzheimer's Disease Neuroimaging Initiative (ADNI) dataset. The ADNI is a longitudinal multicenter consortium of multi‐modal (imaging and non‐imaging) data which started in 2004 and has since grown to include four studies exploring the early detection, intervention, prevention, and treatment of Alzheimer's disease dementia.^33^ The open‐source, longitudinal, and multi‐modal features of this study make it attractive for machine learning research. This reliance and overrepresentation of studies using ADNI poses a limitation on the generalizability of the methods used. ADNI was designed to represent a clinical trial population that is biased toward older ages and more advanced pathology than may be observed population‐wide^34^, ^35^ and is also subject to demographic sampling biases toward socioeconomic status and ethnicity.

Other popular open‐source data sets include the Australian Imaging, Biomarker & Lifestyle Flagship Study of Ageing (AIBL), and the Open Access Series of Imaging Studies (OASIS). These research studies often have strict imaging protocols resulting in highly quality controlled imaging data; however, in hospital and memory clinics data quality can be more variable. Solely relying on large research studies can, therefore, limit generalizability. Fifteen studies utilized in‐house, custom data sets from memory clinics or hospitals. These studies have the advantage of being able to tailor the imaging protocol to the specific study, balance group sizes, and ensure consistency across timepoints. However, due to constraints in recruiting participants, such studies often have smaller sample sizes (*n* = 40 to *n* = 2169), with the largest being a non‐imaging study based on routinely available clinical test scores.

We identified variability in sample size across all included studies with 11 ≤ *n* ≤ 95,202 (*n* = total number of participants across all included data sets). This is important, as increasing the number of examples seen during training can improve performance and robustness to heterogeneity. Twenty‐one studies combined multiple data sets to increase the training power. Sample size is also important for during inference, since larger test sets provide better estimations of performance on unseen data and increased confidence. Forty‐five of 92 studies used a hold‐out test set, either from a subset of the initial data or an additional, independent source. The remaining studies opted for nested, or internal cross‐validation approaches to quantify performance. However, cross‐validation has been shown to underestimate confidence intervals errors, particularly when using small data sets, and is therefore not suitable as a reliable estimate of predictive power.^36^ Only 17 studies used data from external sources to create an independent test set and explicitly test the generalizability of the trained models. Although the performance often drops in external data sets, it can provide a better indication of out‐of‐sample model behavior, which is useful for clinical translation. This is also important for interpretability, as models with generalizable performance are better positioned to distinguish significant, robust important features from noise.

Seventy‐seven of 92 studies used imaging as part of the study, and 59 used imaging alone (one used retinal instead of brain imaging). Some of these (*n* = 28) fed the whole image into the prediction model, whereas others performed voxel‐ or vertex‐wise analysis (*n* = 17), for example, voxel‐based morphometry or extracted regional measures, for example, cortical thicknesses (*n* = 27). Most whole image–based studies utilized 3D data (approximately isotropic voxels or multiple slices per participant, *n* = 21) as opposed to single 2D slices (*n* = 7). We observed a shift toward 3D whole image–based studies with time, likely due to hardware advancements and the increased performance benefits of deep learning over tabular data‐based machine learning methods.

### 4.2 Implementation details

Technical details regarding the choice of predictive model, reported performance, and interpretation across all included studies can be found in Table S2. Here we comment on the observed model accuracies, identified important regions, and the various approaches to validate their explanations.

#### 4.2.1 Model accuracy

For the task of classifying patients with Alzheimer's disease from healthy controls, reported model accuracies ranged from 77.0% to 96.8%. However, Rieke and colleagues^37^ clearly state that “[their] focus was on the different visualization methods and not on optimizing the network,” which may explain the lower reported accuracy values. On the other hand, the highest accuracy of 96.8% (area under the curve [AUC] = 99.6%, *n* = 83) was reported by Qiu and colleagues,^38^ where they used a multi‐modal approach combining a fully convolutional neural network with age, gender, and cognitive scores (Mini‐Mental State Examination [MMSE]). They also evaluate the generalizability of their model using independent, external data sets acquired from the Australian Imaging, Biomarker & Lifestyle Flagship Study of Ageing (AIBL) and the National Alzheimer's Coordinating Center (NACC) and reported accuracy values of 93.2% (AUC = 97.4%, *n* = 382) and 85.2% (AUC = 95.4%, *n* = 565), respectively.

For the more challenging task of identifying individuals with mild cognitive impairment (MCI) who later converted to Alzheimer's disease (pMCI) from those that remained stable (sMCI), the range of reported classification accuracies was 65.4% to 88.5%.^39^, ^40^ The overall drop in performance for this task is expected, since the definition of the MCI label is ambiguous and can differ between centers, leading to a heterogeneous group of participants at various disease stages.^41^ In addition, there is a less‐distinct difference in the neuropathology between these two groups compared to individuals with and without dementia. Therefore, most machine learning studies rely on clinical diagnosis based on symptoms or follow‐up assessments to identify subjects in the MCI group. Nine studies performed multi‐class classification to identify individuals with MCI from healthy controls and dementia patients, or to classify patients with varying degrees of disease severity (e.g., very mild, mild, or moderate dementia).^42^

For tasks involving other phenotypes such as frontotemporal or Parkinson's disease dementia, similar accuracies up to 97.0%^43^, ^44^, ^45^ were found, highlighting the predictive power of machine learning approaches to diagnosis outside of Alzheimer's disease. It is important to note that without performing a thorough meta‐analysis, care must be taken when comparing results due to variability in sample size, validation strategies (whether values are given from cross‐validation or hold‐out test sets), and the amount of time spent optimizing hyperparameters.

#### 4.2.2 Model interpretability

All studies applied interpretability techniques to identify or visualize the most important features. The choice of interpretability method is strongly dependent on the underlying prediction model. Popular approaches included simple visualization or ranking features based on learned weights to more complex occlusion‐based techniques and class‐activation mapping. Although the latter two are more commonly associated with explanations in the image domain, weight visualization or ranking is useful for models such as support vector machines (SVM) or logistic regression, as each weight corresponds to an input feature and can be used to infer their relative importance.

For studies with neuroimaging, this typically involved overlaying heatmaps on a representative brain scan and discussing the regions associated with a specific class. For example, disease probability maps produced by the classification model of Qiu and colleagues identified the temporal lobes, hippocampus, cingulate cortex, corpus collosum, and parts of the parietal and frontal lobes as high risk for classification as an Alzheimer's disease patient.^38^ This was replicated across many of the included studies, with the hippocampus consistently reported as one of the most informative regions (*n* = 43/76). Eight studies did not infer any specific regions from their visualizations or model explanations. For non‐imaging studies, the type of explanations varied due to the different input features and modeling approaches. For example, studies that utilized electronic health records and clinical information (*n* = 5) commonly reported known risk factors such as age, smoking, cardiovascular problems, and lack of exercise as predictive of future dementia diagnosis.

Multi‐modal approaches provide opportunities to investigate where imaging or non‐imaging data were most predictive. Although this depends heavily on the nature of the experiment and data fusion method, such studies demonstrate the utility of including multiple sources of information via increased performance and by ranking the most important features. For example, Venugopalan and colleagues found that the Rey Auditory Verbal Learning Test was a distinguishing feature even in the presence of other imaging‐derived regional features.^46^ Velazquez and colleagues also included this test as a feature but instead found that the 13‐item Alzheimer's Disease Assessment Scale (ADAS) and Functional Activities Questionnaire were more useful, among other factors such as age and hippocampal volume.^47^ However, given that cognitive tests are designed specifically to be used as dementia biomarkers, it is unsurprising that these are highlighted by predictive models for classification tasks. On the other hand, Polsterl and colleagues noted that whilst clinical variables were as relevant as hippocampal shape in most cases, there were a few exceptions amongst Alzheimer's Disease patients, demonstrating subject‐level variability.^48^

For other diagnoses such as frontotemporal dementia, Hu and colleagues identified the right frontal white matter, left temporal, bilateral inferior frontal, and parahippocampal regions as valuable for prediction.^44^ The model used by Morales and colleagues highlighted cerebral white matter and volumes of the lateral ventricles and hippocampi as most relevant to dementia in Parkinson's disease.^45^ Studies that performed differential diagnosis were also able to compare the important regions across dementias. For example, Iizuka and colleagues highlight the significance of the cingulate island sign on brain perfusion single‐proton emission computed tomography (SPECT) imaging for differentiating between subjects with dementia with Lewy bodies and those with dementia of the Alzheimer's type.^49^

#### 4.2.3 Validation approaches

A crucial challenge is how to validate the resulting explanations, particularly in the absence of in vivo ground truth. Most imaging‐based studies (*n* = 41/58) relied on previous research on the neuroanatomic correlates of dementia to qualitatively assess whether their model is utilizing disease‐specific regional information. Seven studies designed statistical tests to quantify the discriminative power of the identified regions of interest or their correlation with other biomarkers and predictors. Both Böhle and colleagues and Dyrba and colleagues correlated the relevance assigned to hippocampus with hippocampal volume; an indicator of atrophy.^50^, ^51^ Bae and colleagues correlated the mean intensity values of identified regions with the rate of change of several measures of cognitive decline.^52^ Others (*n* = 4) used *t*‐tests to compare their findings with traditional analysis methods such as voxel‐based morphometry.^2^, ^51^, ^53^, ^54^ Hu and colleagues performed a *t*‐test to compare the important regions associated with patients with Alzheimer's disease with those associated with frontotemporal dementia.^44^ Liu and colleagues conducted causal analysis using genetic information alongside imaging‐driven important regions.^55^

Three studies made use of simulated data sets, where they had control over the group‐separating features to perform preliminary tests of the explanation method.^56^, ^57^, ^58^ Studies that used multiple interpretability methods (*n* = 9) were also able to comment on whether these highlighted the same regions. Four studies validated their findings by reporting a second classification accuracy using the identified regions of interest as input features^57^, ^59^, ^60^ or incorporating them as anatomic landmarks.^61^

Despite these efforts, most studies were unable to assess the utility of other regions that were highlighted by the model but were without known pathological relevance. Although Qiu and colleagues correlated their findings for 11 subjects with post‐mortem neuropathology, they lacked the statistical power to draw any significant insights.^38^ This challenge also prevailed for non‐imaging studies, although models based on demographic information utilized known risk factors,^62^ and speech‐based models were able to contextualize their findings with phrases and indicators associated with Alzheimer's disease.^63^ Moreover, many of the diagnostic labels in publicly available data sets are based on clinicians’ ratings, which have been shown to be subjective and can be confirmed only through post‐mortem analysis. Therefore, some studies may include dementia patients with mixed pathology, including vascular dementia, which should be considered when assessing potential diagnostic specificity of model predictions.

## 5 DISCUSSION

Our results highlight the growth in this cross‐disciplinary research area, particularly through the combination of neuroimaging and neural networks, which can match and outperform clinical predictions across a range of dementia‐related tasks. The range of accuracies indicate that interpretable models do not necessary require a loss in performance, previously seen as a limitation of IML, as studies have still been able to demonstrate ways to probe the “black‐box,” identify important features, or provide rule‐based explanations.^64^, ^65^ To maximize the impact of machine learning in clinical practice, we provide recommendations to aid clinicians when interpreting results, encourage more homogenous reporting standards, and highlight several challenges that remain.

### 5.1 Recommendations for interpreting interpretability studies

Here we provide recommendations for comprehending studies on IML to help researchers interpret the results accurately:

**Scrutinize the interpretability method details**: Currently all interpretability methods have limitations and drawbacks. Techniques such as occlusion are strongly linked to the sample size, as the more samples seen during training, the more robust it will be to changes in non‐disease relevant patches. Sample size is also important for heterogenous disease pathologies. Data augmentation methods help to build models that generalize well to new cases; however, heterogeneity can make it difficult to decipher between patient‐specific disease relevant pathology and spurious artifacts of the interpretation technique. There is also a strong dependence on model performance. This should be considered when being presented with interpretability findings, as explanations from models with poor predictive power may be inaccurate and group‐level findings are likely to be affected by falsely classified samples.

**Identify whether the method is model‐, group‐, or individual‐level**: The results can differ greatly depending on whether the output is group‐level or individual‐level, and the pathways to clinical impact will vary as a result. Occlusion techniques are often not suitable for making individual‐level explanations for a given prediction. The results obtained by occluding patches across a single example case are still a representation of the overall model susceptibility to a given patch. In contrast, methods such as LRP and CAM allow for individual heatmaps that reflect the regional relevance associated with a single case.

**Relevance and importance do not guarantee biological significance**: Although interpretable methods present exciting opportunities to improve our understanding of model predictions, the results are not necessarily related to biological or pathological features. Many of these methods are model agnostic or have been developed primarily outside the medical imaging context. Therefore, they lack considerations of causation needed to correlate their outputs with biological relevance. The values and scores derived from methods such as LRP are better interpreted as “where the model sees evidence” ^50^ or in the case of class activation maps, “which features has the model learned as relevant to this class.” However, they are not sufficient for identifying potential interactions between voxels or features, or high‐level concepts such as atrophy. Similarly, some identified features may be a result of the presence of noise, artifacts, or group differences from the underlying data, which can be misleading.

### 5.2 Recommendations for study design and report writing

When carrying out studies that incorporate interpretable methods, we highlight three recommendations when (designing the experiment and) reporting their findings:

**Design the entire study with the end‐user in mind**: The choice of interpretability method depends on the needs of the end‐user. Therefore, it can be beneficial to conceptualize the type of questions to be asked, whether that may be “which features are most important to the model” or “for this individual, how have the input features been used to arrive at the final prediction?” Addressing the interpretability of the study early on will allow researchers to better design their study, such as determining whether ground‐truth annotations may be desired to validate their interpretability models or if simulated preliminary results could benefit them as previously seen.^56^, ^58^, ^66^ Research aiming to perform classification between disease groups may be better suited toward group‐level post hoc explanations that are able to highlight specific features of interest. Alternatively, if the focus of the research is to better understand the disease‐causing pathology, then counterfactual examples that provide clinicians with an explanation of which brain changes would convert a diagnosis from healthy to dementia may be more useful.

**Use diverse data sets**: Our review identified a strong bias toward certain data sets in data‐driven approaches for dementia research, such as the ADNI (Alzheimer's Disease Neuroimaging Initiative). To better understand the limitations and potential application for both interpretable methods and the predictive models themselves, it is important to use data derived from different cohorts and different acquisition methods. For example, in Etminani and colleagues,^43^ data across multiple studies was used to evaluate a model using individuals with Alzheimer's disease, dementia with Lewy bodies, and frontotemporal dementia such that they could evaluate the model's generalizability. Although open‐source data sets are crucial for the development of robust predictive models, they do not always provide a reliable measure of performance in a clinical setting, where image quality may be poorer, sample sizes are smaller, and cohorts may be more demographically diverse. Extending research in this area to clinically acquired data sets could also create opportunities to explore and identify bias by observing differences in model explanations across groups.

**Consistently adhere to reporting standards**: Adherence to reporting standards will play a crucial role in the development of this field as researchers will be able to quantitatively compare performance across studies (e.g., meta‐analyses) and better contextualize results. Although several checklists and guidelines such as CLAIM (Checklist for Artificial Intelligence in Medical Imaging)^67^ and STARD (Standards for Reporting of Diagnostic Accuracy Studies)^68^ exist for AI applications in health care, here we emphasize areas in which we observed large variability across the included studies. For example, when reporting model performance results, we suggest that researchers provide confusion matrices, as they provide concise access to several measures of performance such as balanced accuracy, sensitivity, and specificity. Single measures of accuracy may not be sufficient, particularly in dementia studies where unbalanced data sets are common, and sensitivity to true positive cases may be more desirable than robustness to false positives. We also re‐emphasize the importance of clearly specifying the sample size across prediction tasks and data sets and providing confidence intervals where available. This amount of detail varied among the studies included in our review but is important, particularly when reporting results from multiple prediction tasks. Data sets also differ in their labeling procedures, so studies must be careful when training models across cohorts and clearly highlight any discrepancies. Many dementia‐causing diseases can only truly be diagnosed post‐mortem, and definitions of categories such as mild cognitive impairment are still debated.^69^ Furthermore, in imaging studies where multiple scans are available per participant (i.e., from several time points), researchers should ensure that their methods are robust to data leakage by splitting their data sets on a subject level and clearly stating if multiple scans have been used during training or testing. Models should be tested on hold‐out test sets (and external data sets where possible) rather than relying on cross‐validation for more a reliable estimate of performance on new data.

### 5.3 Remaining challenges

A key challenge that remains is that IML methods have yet to be thoroughly tested to ensure that they are robust and reliable. Some research efforts in computer vision have attempted to address this. For example, Adebayo and colleagues define and tested several post hoc explanation methods against pre‐defined sanity checks to see if explanations were robust to small perturbations in the data and different architectures.^70^, ^71^ Several methods failed these tests and were deemed to be unreliable. Moreover, Tian and colleagues evaluated the test‐retest reliability of feature importance for models trained to predict cognition, and they elucidated a trade‐off between feature weight reliability and model performance.^72^ Our review identified one study, which assessed the robustness of two explanation methods by defining a continuity and selectivity metric. In that study, the authors tested whether the heatmaps produced via perturbation and occlusion techniques are consistent across similar images (continuity) and whether relevant occluded regions correlated with the change in class probability (selectivity).^73^ They also quantitatively compared the heatmaps and their robustness characteristics across different model architectures. A similar test was carried out by Thibeau‐Sutre and colleagues who compared heatmaps produced across multiple cross‐validation folds as well as different hyperparameter values.^74^ However, none of these measures consider characteristics that are application specific, such as robustness to scanner artifacts or non–disease‐related variability in brain structure that may arise in more clinical, diverse data sets. Moreover, a particular explanation method may be insufficient according to the test defined in computer vision–based studies but may still be sufficient for decision support. Context‐specific quality criteria is needed to ensure that the outputs are clinically useful, while affording some flexibility against strict test as the field of IML continues to develop.

There was also a limited involvement of neuroradiologists and clinicians throughout these studies. This is essential to designing informed experiments that address the relevant questions and ensuring that work in this field has an impact on translation. Ding and colleagues used radiologists at the diagnostic level to demonstrate whether the deep learning model outperforms on an independent test set.^75^ However, none of the studies identified through our review incorporated clinicians to systematically validate model explanations. Although there is a range of supporting literature, perspectives and reviews highlighting the need for interpretable machine learning in medical imaging,^11^, ^27^, ^29^, ^76^, ^77^ being able to demonstrate its impact through semi‐structured interview and qualitative analysis would be a key step toward proving how such techniques can fulfil it. Moreover, the complexity and heterogeneity of neurogenerative disease pathology has limited researchers’ ability to make conclusive statements about newly identified regions of interest. The lack of expert validation meant that studies rely on comparisons to previous literature, as discussed in Section 4.2.2. This creates a potential contradiction that can inhibit the discovery of new mechanistic insights. Therefore, a challenge lies in finding balance between designing experiments that can systematically evaluate and quantify the accuracy of model explanations, while also being able to identify clinically useful biomarkers from the results.

### 5.4 Future directions

Interpretable machine learning has the potential to enhance the dementia prediction pipeline and open avenues for new insights into disease mechanisms. Group‐ or patient‐level explanations could be useful for identifying features that are relevant to specific phenotypes or stages and aiding the development of preventative therapies. Identifying which regions the model focuses on could also be used to influence other stages in the imaging protocol. For instance, acquisition sequences could be optimized for imaging‐specific regions of interest, even in real time.^78^ More generally, being able to differentiate between biologically relevant features specific to groups with similar clinical profiles helps to demonstrate the benefit of computer‐assistive technologies. Individualized, patient‐specific explanations can serve as a huge step toward personalized medicine with clinicians being able to identify key drivers of a patient's diagnosis. Looking ahead, interpretable models could help to advance scientific discovery by identifying novel biomarkers such as disease‐specific genes.^79^ Although machine learning is not currently used in clinical trial recruitment, model explanations also provide opportunities to enhance patient stratification or explore treatment response through predictors associated with specific brain regions.

## 6 CONCLUSION

Interpretability is key for the clinical application of machine learning in decision‐making tools for dementia prediction. The need for model explanations has been identified both in the legal sector and health services as the use of machine learning based solutions continues to rise. In this systematic review, three databases were searched to identify 92 studies that have applied interpretable methods to machine learning models designed for the prediction of dementia. We found a large bias toward open‐source data sets such as ADNI, which may have limited the generalizability of findings. A key emerging theme was the challenge of validating interpretation methods. Although this challenge also exists outside of dementia research, we highlight that domain‐specific quality criteria may also require critical assessment of the clinical utility. Dementia prediction tasks are made ever more difficult by the high dimensionality of data and interactions between factors such as age, sex, genetic history, and lifestyle. Building models that make use of this multi‐modal landscape of information but can still disentangle their influences on the output would help bring the power of machine learning models one step closer to large‐scale clinical adoption.

## AUTHOR CONTRIBUTIONS

**Sophie A. Martin**: Implementation, data extraction, figure generation, paper writing. **Florence J. Townend**: Data extraction, paper review. **James H. Cole**: Conceptualization, study supervision, paper review. **Frederik Barkhof**: Study supervision, paper review.

## CONFLICTS OF INTEREST

Dr Barkhof reports board membership from Neurology, board membership from Radiology, board membership from the Medical Science Journal, board membership from Neuroradiology, personal fees from Springer, personal fees from Biogen, grants from Roche, grants from Merck, grants from Biogen, personal fees from IXICO, grants from European Innovative Medicines Initiative, grants from GE Healthcare, grants from the UK Multiple Sclerosis Society, grants from the Dutch Multiple Sclerosis Research Foundation, grants from Nederlands Wetenschappelijk Onderzoek, grants from the National Institute for Health and Care Research, personal fees from Combinostics, and personal fees from Prothena, outside the submitted work; and is co‐founder and stock owner of Queen Square Analytics. The other authors have no relevant conflicts of interest to disclose.

## CONSENT STATEMENT

Consent was not necessary for this work.

## Supporting information

Supporting Information
