# Supplementary material for: Interpretable machine learning for dementia: A systematic review
Source: Alzheimers Dement. 2023 Feb 3;19(5):2135–49. doi: 10.1002/alz.12948 (PMC10955773; doi:10.1002/alz.12948)
Supplement: Supplementary file 1 — Supporting Information [file ALZ-19-2135-s001.docx]

**Supplementary Material**

**Supplementary Table 1**: Study details for all included papers including the modalities used, data sources, diagnostic categories, total number of participants included in the study (summed over all data sources), type of input fed to the predictive model, whether data augmentation was used and whether the code is open source.

*Abbreviations. MRI = magnetic resonance imaging (refers to T1-weighted imaging otherwise stated), PET = positron emission tomography, Blood-based = blood-based biomarkers, Dem. = demographic data, fMRI = functional magnetic resonance, CSF = cerebrospinal fluid, SPECT = single-photon emission computed tomography, DTI = diffusion tensor imaging, EEG = electroencephalogram, OCT = optical computed tomography, AD = Alzheimer’s disease, DLB = dementia with Lewy Bodies, FTLD = frontotemporal dementia, [e/l/s/p/a]MCI = [early/late/stable/progressive/amnestic]mild cognitive impairment, CN = cognitively normal, -C = converted, -NC = not converted, iNPH = idiopathic normal pressure hydrocephalus, SD = severe dementia, MD = mild dementia, ND = no dementia, MCI_AD = mild cognitive impairment converted to AD, SMC = significant memory concern, MID = memory impairment disorders, VCD = vascular cognitive disorders, DWS = dementia with stroke, PD[D/CI/MCI] = Parkinson’s disease [dementia/cognitively intact/mild cognitively impaired].*

| Author, Year | Datatype(s) | Dataset(s) | Labels | Total # of Participants | Model Input | Data Aug. | Open source |
| --- | --- | --- | --- | --- | --- | --- | --- |
| Abrol, 2020[^1^](#_ENREF_1) | MRI + Dem. | ADNI | AD, MCI, CN | 828 | 3D-map | Yes | No |
| Abuhmed, 2021[^2^](#_ENREF_2) | MRI + PET + Dem. + Clin. | ADNI | AD, sMCI, pMCI, CN | 1371 | Vector | No | No |
| Achilleos, 2020[^3^](#_ENREF_3) | MRI | ADNI | AD, CN | 213 | Extracted brain features | Yes | No |
| Adel, 2017[^4^](#_ENREF_4) | MRI | ADNI, OASIS | AD, sMCI, pMCI, CN | 1017 | Vector | No | No |
| Azcona, 2020[^5^](#_ENREF_5) | MRI | ADNI | AD, CN | 435 | Cortical mesh | No | No |
| Bae, 2021[^6^](#_ENREF_6) | MRI | ADNI | AD, MCI-C, MCI-NC, CN | 3940 | 3D-whole-brain | No | No |
| Balagopalan, 2021[^7^](#_ENREF_7) | Speech | ADReSS | AD, CN | 156 | Vector | No | No |
| Bang, 2017[^8^](#_ENREF_8) | EHR + Dem. + Clin. | CREDOS | Dementia, CN | 14917 | Vector | No | No |
| Beebe-Wang, 2021[^9^](#_ENREF_9) | Dem. + Clin. | ROSMAP | AD, CN | 1597 | Vector | No | Yes |
| Ben Miled, 2020[^10^](#_ENREF_10) | EHR + Dem. + Clin. | EMR | Dementia, CN | 13717 | Vector | No | No |
| Bloch, 2021[^11^](#_ENREF_11) | MRI + Dem. | ADNI, AIBL | sMCI, pMCI | 747 | Vector | No | No |
| Bloch, 2021[^12^](#_ENREF_12) | MRI + Dem. + Clin. | ADNI, AIBL | AD, sMCI, pMCI, CN | 2312 | Vector | No | No |
| Böhle, 2019[^13^](#_ENREF_13) | MRI | ADNI | AD, CN | 344 | 3D-whole-brain | Yes | Yes |
| Choi, 2020[^14^](#_ENREF_14) | PET | ADNI, UKPD BB | AD, PD, sMCI, CN | 636 | 3D-whole-brain | No | No |
| Chyzhyk, 2014[^15^](#_ENREF_15) | MRI | OASIS | AD, CN | 98 | Extracted brain features | No | No |
| Cuingnet, 2013[^16^](#_ENREF_16) | MRI | ADNI | AD, CN | 299 | 3D-map, Extracted brain features | No | No |
| Danso, 2021[^17^](#_ENREF_17) | Dem. + Clin. | SHARE, PREVENT | AD, CN, High Risk, Low Risk | 84856 | Vector | No | No |
| Das, 2019[^18^](#_ENREF_18) | CSF + Blood-based | ADNI | AD, CN | 151 | Vector | No | No |
| Ding, 2019[^19^](#_ENREF_19) | PET | ADNI, In-house | AD, MCI, CN | 1042 | 2D-slices | Yes | No |
| Ding, 2022[^20^](#_ENREF_20) | MRI | ADNI, Peking University Third Hospital of China | AD, CN | 434 | Extracted brain features | No | No |
| Doborjeh, 2021[^21^](#_ENREF_21) | MRI | MAS | AD, MCI, CN | 175 | Vector | No | No |
| Donini, 2019[^22^](#_ENREF_22) | MRI + Dem. | ADNI | AD, MCI | 227 | Vector | No | Yes |
| Durongbhan, 2019[^23^](#_ENREF_23) | EEG | Sheffield Teaching Hospitals Memory Clinic | AD, CN | 40 | Vector | Yes | No |
| Dyrba, 2020[^24^](#_ENREF_24) | MRI | ADNI | AD, MCI, CN | 662 | 3D-map | Yes | No |
| Dyrba, 2021[^25^](#_ENREF_25) | MRI + PET | ADNI, AIBL, DELCODE | AD, MCI, CN | 2318 | 3D-map | Yes | Yes |
| Eitel, 2019[^26^](#_ENREF_26) | MRI | ADNI | AD, CN | 344 | 3D-whole-brain | Yes | No |
| El-Sappagh, 2021[^27^](#_ENREF_27) | MRI + PET + CSF + Dem. + Clin. | ADNI | AD, sMCI, pMCI, CN | 1048 | Vector | No | No |
| Esmaeilzadeh, 2018[^28^](#_ENREF_28) | MRI + Dem. | ADNI | AD, MCI, CN | 841 | 3D-whole-brain | Yes | No |
| Etminani, 2021[^29^](#_ENREF_29) | PET | ADNI, ELDB, FTLDNI | AD, MCI-AD, DLB, FTLD, CN | 757 | 3D-map | No | No |
| Fan, 2021[^30^](#_ENREF_30) | MRI | ADNI, AIBL | AD, eMCI, lMCI, CN | 204 | 3D-whole-brain | No | No |
| Ford, 2020[^31^](#_ENREF_31) | EHR | UK CPRD | AD, VD, CN | 95202 | Vector | No | No |
| Gu, 2021[^32^](#_ENREF_32) | fMRI | ADNI | AD, MCI, CN | 311 | Connectivity matrix | No | No |
| Guan, 2022[^33^](#_ENREF_33) | MRI | ADNI | AD, pMCI, sMCI, CN | 1340 | 3D-whole-brain | Yes | No |
| Gutierrez-Becker, 2018[^34^](#_ENREF_34) | MRI | ADNI | AD, MCI, CN | 7974 | Vector | Yes | No |
| He, 2021[^35^](#_ENREF_35) | MRI + DTI + fMRI | ADNI | AD, MCI, CN | 1139 | 3D-whole-brain | Yes | No |
| Hu, 2021[^36^](#_ENREF_36) | MRI | ADNI, NIFD | AD, FTD, CN | 1797 | 3D-whole-brain | Yes | Yes |
| Huang, 2021[^37^](#_ENREF_37) | MRI | ADNI, OASIS, AIBL | AD, MCI, CN | 416 | 3D-whole-brain | No | No |
| Iizuka, 2019[^38^](#_ENREF_38) | SPECT | In-house | AD, DLB, CN | 300 | 2D-slices | Yes | No |
| Irie, 2020[^39^](#_ENREF_39) | MRI | In-house | AD, INPH, CN | 69 | 3D-map | No | No |
| Jain, 2021[^40^](#_ENREF_40) | MRI | Kaggle | SD, MD, MCI, CN | 5119 | 2D-slices | Yes | No |
| Jin, 2019[^41^](#_ENREF_41) | MRI | ADNI | AD, CN | 532 | 3D-map | No | No |
| Jin, 2020[^42^](#_ENREF_42) | MRI | ADNI, In-house | AD, MCI, CN | 1832 | 3D-map | No | No |
| Jo, 2020[^43^](#_ENREF_43) | PET | ADNI | AD, MCI, CN | 300 | 3D-map | Yes | No |
| Kamal, 2021[^44^](#_ENREF_44) | Genetics | NCBI | AD, CN | 104 | Vector | No | No |
| Khan, 2019[^45^](#_ENREF_45) | MRI | ADNI | AD, MCI, CN | 150 | 2D-slices | No | Yes |
| Kroll, 2020[^46^](#_ENREF_46) | MRI | ADNI | AD, MCI-C, MCI-NC, CN | 726 | Vector | No | No |
| Lee, 2019[^47^](#_ENREF_47) | MRI | ADNI | AD, MCI, CN | 801 | 3D-map | No | No |
| Lee, 2021[^48^](#_ENREF_48) | PET | ADNI, CARPET | AD, CN, Pre-stroke Dementia, Post-stroke Dementia, Non-dementia | 802 | 3D-whole-brain | No | No |
| Li, 2018[^49^](#_ENREF_49) | Clinical | In-house | AD, MCI, CN | 729 | Vector | No | No |
| Li, 2021[^50^](#_ENREF_50) | MRI | ADNI | AD, CN | 560 | 3D-whole-brain | No | No |
| Lian, 2020[^51^](#_ENREF_51) | MRI | ADNI | AD, sMCI, pMCI, CN | 821 | 3D-patches | Yes | No |
| Liu, 2019[^52^](#_ENREF_52) | DTI + Genetics | ADNI | AD, MCI, CN | 151 | 3D-whole-brain | Yes | No |
| Liu, 2021[^53^](#_ENREF_53) | MRI | ADNI | AD, CN | 414 | 3D-whole-brain | No | Yes |
| Mendoza-Léon, 2020[^54^](#_ENREF_54) | MRI | OASIS | AD, CN | 174 | 2D-patches | No | No |
| Moon, 2021[^55^](#_ENREF_55) | Genetics | ANM Consortium, MRC Brain Bank | AD, MCI, CN | 793 | Vector | No | No |
| Morabito, 2021[^56^](#_ENREF_56) | EEG | IRCCS Centro Neurolesi Bonino Pulejo of Messina | AD, MCI | 11 | Vector | No | No |
| Morales, 2013[^57^](#_ENREF_57) | MRI | In-house | PDCI, PDMCI, PDD | 45 | Vector | No | No |
| Murugan, 2021[^58^](#_ENREF_58) | MRI | Kaggle | Mild, Mod, Very Mild, CN | 12800 | 2D-slices | Yes | Yes |
| Nigri, 2020[^59^](#_ENREF_59) | MRI | ADNI, AIBL | AD, CN | 826 | 3D-patches | No | No |
| Orlenko, 2021[^60^](#_ENREF_60) | SNP | ADNI | AD, MCI, CN | n/a | Vector | No | No |
| Ortiz, 2016[^61^](#_ENREF_61) | MRI | ADNI | AD, eMCI, lMCI, CN | 275 | Extracted brain features | No | No |
| Ortiz-Suárez, 2017[^62^](#_ENREF_62) | MRI + Clin. | OASIS | AD, CN | 86 | 2D-slices | No | No |
| Pohl, 2021[^63^](#_ENREF_63) | MRI | ADNI | AD, CN | 1447 | 3D-whole-brain | No | No |
| Polsterl, 2021[^64^](#_ENREF_64) | MRI + Clin. | ADNI | AD, CN | 1653 | Point cloud | No | No |
| Qiu, 2020[^65^](#_ENREF_65) | MRI + Dem. + Clin. | ADNI, AIBL, NACC, FHS | AD, CN | 1466 | Extracted brain features | No | Yes |
| Ren, 2019[^66^](#_ENREF_66) | MRI | ADNI | AD, MCI, CN | 692 | 2D-masked-image | Yes | No |
| Rieke, 2018[^67^](#_ENREF_67) | MRI | ADNI | AD, CN | 344 | 3D-whole-brain | No | Yes |
| Ronge, 2021[^68^](#_ENREF_68) | MRI + CSF + Dem. + Genetics | ADNI | AD, MCI, CN | 1492 | Vector | No | No |
| Ruiz, 2018[^69^](#_ENREF_69) | MRI | ADNI | AD, sMCI, CN | 818 | 3D-patches | No | Yes |
| Sarica, 2021[^70^](#_ENREF_70) | MRI | ADNI | sMCI, pMCI | 200 | Extracted brain features | No | No |
| Sha, 2021[^71^](#_ENREF_71) | Blood-based | In-house | AD, aMCI, CN | 172 | Vector | No | Yes |
| Shahamat, 2020[^72^](#_ENREF_72) | MRI | ADNI | AD, CN | 140 | 3D-masked-image | No | No |
| Shaji, 2021[^73^](#_ENREF_73) | MRI | ADNI | AD, CN | 200 | 2D-slices | Yes | No |
| Shoaip, 2021[^74^](#_ENREF_74) | MRI + PET + CSF + Dem. + Clin. | ADNI | AD, eMCI, sMCI, lMCI, CN | 2256 | Vector | No | No |
| Song, 2021[^75^](#_ENREF_75) | MRI + PET | ADNI | AD, MCI, CN | 381 | 3D-whole-brain | No | No |
| Song, 2021[^76^](#_ENREF_76) | PET | ADNI | n/a | n/a | Extracted brain features | No |  |
| Souillard-Mandar, 2016[^77^](#_ENREF_77) | Clinical | In-house, FHS | MID, VCD, PD, CN | 2169 | Vector | No | No |
| Sun, 2018[^78^](#_ENREF_78) | MRI | ADNI | AD, sMCI, pMCI, CN | 1328 | Extracted brain features | No | Yes |
| Thibeau-Sutre, 2020[^79^](#_ENREF_79) | MRI | ADNI, AIBL | AD, CN | 1171 | 3D-whole-brain | No | No |
| Tian, 2021[^80^](#_ENREF_80) | OCT | UK Biobank | AD, CN | 174 | Vector | No | No |
| Vandenberghe, 2013[^81^](#_ENREF_81) | MRI + PET | ALZ201 Phase 2 Study | AD, CN | 112 | 3D-map | No | No |
| Velazquez, 2021[^82^](#_ENREF_82) | MRI + PET + Dem. + Clin. | ADNI | eMCI-C, eMCI-NC | 383 | Vector | No | No |
| Venugopalan, 2021[^83^](#_ENREF_83) | MRI + Dem. + Clin. | ADNI | AD, CN | 2004 | Extracted brain features | No | No |
| Vigneron, 2016[^84^](#_ENREF_84) | PET | ADNI | AD, sMCI, pMCI, CN | 335 | 3D-patches | No | No |
| Wehenkel, 2017[^85^](#_ENREF_85) | MRI + PET | OASIS, In-house | MCIc, MCIs, Dementia, NC | 145 | Extracted brain features | No | No |
| Wehenkel, 2018[^86^](#_ENREF_86) | MRI + PET + Dem. + Clin. | In-house | sMCI, pMCI | 45 | Vector | No | No |
| Yang, 2018[^87^](#_ENREF_87) | MRI | ADNI | AD, CN | 103 | 3D-whole-brain | No | No |
| Yee, 2020[^88^](#_ENREF_88) | PET | ADNI | sNC, sDAT, uNC, pNC, sMCI, pMCI, eDAT | 606 | 3D-whole-brain | Yes | No |
| Zeng, 2020[^89^](#_ENREF_89) | Dem. + Clin. | ADNI | AD, MCI, CN | 543 | Vector | No | No |
| Zhang, 2021[^90^](#_ENREF_90) | MRI + Clin. | ADNI, AIBL, NACC | AD, CN | 1349 | 3D-patches | No | No |
| Zhang, 2021[^91^](#_ENREF_91) | MRI | ADNI | AD, MCI, CN | 1407 | 3D-whole-brain | No | No |
| Zhu, 2022[^92^](#_ENREF_92) | MRI | ADNI | AD, sMCI, pMCI, CN | 202 | Extracted brain features | No |  |

**Supplementary Table 2**: Model and available performance results of included studies on validation/test data. We report the model type (best performing if multiple models were used), predictive tasks, reported accuracy (ACC) /area under the receiver-operator curve (AUC), identify where the quoted values were from i) cross-validation test folds ii) a hold-out test set or iii) external test set. If ii) or iii) we also provide the number of test cases (*italics* indicate that this value has been calculated based on a percentage split where the value was not clearly provided, e.g., if 20% of the total number of subjects were used as a hold-out test set and [brackets] indicate performance ranges estimated from graphs if exact numbers were not provided in the full-text). We also include the IML method used to generate model explanations and specify whether the study attempted to validate the resulting explanations (e.g., comparing to identified regions of interest from alternative methods or simulation). Note that referring to known regions of interest from previous literature was not sufficient.

*Abbreviations. [C/D/S/R]NN = [convolutional/deep/spiking/recurrent]neural network, [F/G)]N = [fully/graph] convolutional network, DBN = deep belief network, SVM = support vector machine, RF = random forest, ET = extremely randomised trees, MKL = multi-kernel learning, MLP = multi-layer perceptron, LGP = linear genetic programming, BiLSTM = bidirectional long short-term memory, SPHOG = spatial pyramid histogram of oriented gradient, GA= genetic algorithm, NB = Naïve Bayes, AD[D] = Alzheimer’s disease [Dementia], DWS = Dementia with stroke, DLB = dementia with Lewy Bodies, FTLD = frontotemporal dementia, MCI = mild cognitive impairment, CN = cognitively normal, iNPH = idiopathic normal pressure hydrocephalus, SD = severe dementia, MD = mild dementia, ND = no dementia, MCI_AD = mild cognitive impairment converted to AD, SMC = significant memory concern, DWS = dementia with stroke, PD[D/CI/MCI] = Parkinson’s disease [dementia/cognitively intact/mild cognitively impaired], VCD = vascular cognitive disorders, DAT = Dementia of Alzheimer’s type, s- = stable, p- = progressive, e- = early, l- = late, a- = amnestic, -C = converted, -NC = not converted, LOOCV = leave-one-out cross validation, CAM = class activation mapping, LRP = layer-wise relevance propagation, LIME = local interpretable model explanations.*

† = balanced accuracy

| Author, Year | Model(s) | Task(s) | ACC (%) | AUC (%) | Test Data | N (#) | IML Method | + Validation |
| --- | --- | --- | --- | --- | --- | --- | --- | --- |
| Abrol, 2020[^1^](#_ENREF_1) | ResNet | AD vs CN | 89.3 | 94.0 | 5-fold CV |  | Occlusion | No |
|  |  | pMCI vs CN | 86.5 | 90.0 |  |  |  |  |
|  |  | AD vs sMCI | 87.5 | 90.0 |  |  |  |  |
|  |  | sMCI vs pMCI | 75.1 | 78.0 |  |  |  |  |
| Abuhmed, 2021[^2^](#_ENREF_2) | BiLSTM + RF | AD vs MCI vs CN | 82.6 |  | Hold-out Test | *137* | Rule extraction | No |
| Achilleos, 2020[^3^](#_ENREF_3) | Decision Trees | AD vs CN | 91.0 |  | 10-fold CV |  | Rule extraction | No |
| Adel, 2017[^4^](#_ENREF_4) | Scattering Transform + SVM | AD vs CN | 84.9 |  | Hold-out Test | *167* | Weight visualisation | No |
|  |  | AD vs CN (OASIS) | 73.0 |  |  | 36 |  |  |
|  |  | sMCI vs pMCI | 73.5 |  |  | *53* |  |  |
| Azcona, 2020[^5^](#_ENREF_5) | GCN | AD vs CN | 96.4 |  | Hold-out Test | *87* | Grad-CAM | No |
| Bae, 2021[^6^](#_ENREF_6) | ResNet | MCI-C vs MCI-NC | 82.4 | 82.7 | Hold-out Test | 175 | Occlusion | Yes |
| Balagopalan, 2021[^7^](#_ENREF_7) | BERT | AD vs CN | 83.3 | 93.0 | 10-fold CV |  | Attention values | No |
| Bang, 2017[^8^](#_ENREF_8) | SVM | Dementia vs CN | 90 | 96.0 | Hold-out Test | *4475* | Rule extraction | No |
| Beebe-Wang, 2021[^9^](#_ENREF_9) | XGBoost | Dementia vs CN | 89.8 | 89.8 | Hold-out Test | 1805 | Shapley values | Yes |
|  |  | Dementia vs CN (ROS) |  | 89.5 | External Test | 1156 |  |  |
|  |  | Dementia vs CN (MAP) |  | 87.9 |  | 649 |  |  |
| Ben Miled, 2020[^10^](#_ENREF_10) | RF | Dementia vs CN | 77 |  | 5-fold CV |  | Selected features | No |
| Bloch, 2021[^11^](#_ENREF_11) | RF | sMCI vs pMCI (ADNI) | 68.5 |  | Hold-out Test | 144 | Shapley values | No |
|  |  | sMCI vs pMCI (AIBL) | 77.5 |  | External Test | 28 |  |  |
| Bloch, 2021[^12^](#_ENREF_12) | RF | AD vs CN (AIBL) | 88.5 |  | External Test | 518 | Shapley values | No |
|  |  | MCI vs AD (AIBL) | 58.8 |  |  | 165 |  |  |
|  |  | CN vs MCI (AIBL) | 48.6 |  |  | 541 |  |  |
|  |  | sMCI vs pMCI (AIBL) | 79.4 |  |  | 34 |  |  |
| Böhle, 2019[^13^](#_ENREF_13) | CNN | AD vs CN | 88 |  | Hold-out Test | 60 | LRP | Yes |
| Choi, 2020[^14^](#_ENREF_14) | CNN | AD vs CN |  | 94.0 | 10-fold CV |  | Grad-CAM | No |
|  |  | MCI-C vs MCI-NC |  | 82.0 | External Test | 441 |  |  |
|  |  | PD vs PDD |  | 81.0 | External Test | 62 |  |  |
| Chyzhyk, 2014[^15^](#_ENREF_15) | GA-MLP | AD vs CN | 86.0 |  | 10-fold CV |  | Selection frequency | Yes |
| Cuingnet, 2013[^16^](#_ENREF_16) | SVM | AD vs CN | 91.0 |  | Hold-out Test | 149 | Weight visualisation | No |
| Danso, 2021[^16^](#_ENREF_16) | XGBoost | AD vs CN (SHARE) | 87.0 | 96.0 | Hold-out Test | 16455 | Shapley values | No |
|  |  | AD vs CN (PREVENT) | 56.5 | 63.0 |  | 85 |  |  |
| Das, 2019[^18^](#_ENREF_18) | Decision Trees | AD vs CN | 90 | 81.0 | 5-fold CV |  | Rule extraction | Yes |
| Ding, 2019[^19^](#_ENREF_19) | InceptionV3 | AD |  | 92.0 | Hold-out Test | 103 | Backpropagation | No |
|  |  | MCI |  | 63.0 |  |  |  |  |
|  |  | CN |  | 73.0 |  |  |  |  |
|  |  | AD (In-house) |  | 98.0 | External Test | 40 |  |  |
|  |  | MCI (In-house) |  | 52.0 |  |  |  |  |
|  |  | CN (In-house) |  | 84.0 |  |  |  |  |
| Ding, 2022[^20^](#_ENREF_20) | SPHOG | AD vs CN | 85.9 |  | Not reported |  | Selection frequency, Accuracy map | No |
| Doborjeh, 2021[^21^](#_ENREF_21) | SNN | Classification | 95.0 |  | Not reported |  | Weight visualisation | No |
|  |  | 2-year ahead prediction | 91.0 |  |  |  |  |  |
|  |  | 4-year ahead prediction | 73.0 |  |  |  |  |  |
| Donini, 2019[^22^](#_ENREF_22) | MKL | AD vs CN | 86.9† |  | 10-fold CV |  | Weight visualisation | No |
| Durongbhan, 2019[^23^](#_ENREF_23) | KNN | AD vs CN | 83.4 |  | 10-fold CV |  | Weight visualisation | No |
| Dyrba, 2020[^24^](#_ENREF_24) | CNN | ADD vs CN | 75.2 | 93.0 | Nested 10-fold CV |  | LRP | Yes |
| Dyrba, 2021[^25^](#_ENREF_25) | CNN | AD vs CN (ADNI-3) | 84.4 | 91.3 | External Test | 388 | LRP | Yes |
|  |  | AD vs CN (AIBL) | 85.0 | 95.0 |  | 510 |  |  |
|  |  | AD vs CN (DELCODE) | 85.5 | 95.3 |  | 319 |  |  |
|  |  | MCI vs CN (ADNI-3) | 63.1 | 68.4 |  | 513 |  |  |
|  |  | MCI vs CN (AIBL) | 68.2 | 76.3 |  | 544 |  |  |
|  |  | MCI vs CN (DELCODE) | 71.0 | 77.5 |  | 370 |  |  |
| Eitel, 2019[^26^](#_ENREF_26) | CNN | AD vs CN | 86.8† |  | Hold-out Test | 60 | Gradient*input, Backpropagation. LRP, Occlusion | No |
| El-Sappagh, 2021[^27^](#_ENREF_27) | RF | AD vs MCI vs CN | 93.4 |  | 10-fold CV |  | Shapley values, Rule extraction | No |
|  |  | sMCI vs pMCI | 87.8 | 95.3 |  |  |  |  |
| Esmaeilzadeh, 2018[^28^](#_ENREF_28) | CNN | AD vs CN | 94.1 |  | 10-fold CV |  | Occlusion | No |
|  |  | AD vs MCI vs CN | 61.0 |  |  |  |  |  |
| Etminani, 2021[^29^](#_ENREF_29) | VGG16 | AD |  | 76.4 | Hold-out Test | 73 | Occlusion | No |
|  |  | MCI-AD |  | 71.4 |  |  |  |  |
|  |  | DLB |  | 96.2 |  |  |  |  |
|  |  | CN |  | 94.7 |  |  |  |  |
| Fan, 2021[^30^](#_ENREF_30) | CNN | AD vs CN | 95.7 |  | 5-fold CV |  | Grad-CAM | Yes |
|  |  | AD vs CN (AIBL) | 85.4 |  | External Test | 23 |  |  |
|  |  | CN vs eMCI | 88.0 |  | 5-fold CV |  |  |  |
|  |  | eMCI vs lMCI | 90.1 |  | 5-fold CV |  |  |  |
|  |  | AD vs lMCI | 90.1 |  |  |  |  |  |
|  |  | AD vs eMCI vs lMCI vs CN | 86.5 |  |  |  |  |  |
| Ford, 2020[^31^](#_ENREF_31) | RF | AD vs CN |  | 89.0 | Hold-out Test | *31417* | Weight values | No |
|  |  | VD vs CN |  | 85.0 |  |  |  |  |
| Gu, 2021[^32^](#_ENREF_32) | GCN | AD vs CN | 89.9 | 91.0 | Hold-out Test | *22* | Occlusion | No |
|  |  | MCI vs CN | 86.3 | 84.0 |  | *20* |  |  |
|  |  | AD vs MCI | 92.0 | 93.0 |  | *21* |  |  |
| Guan, 2022[^33^](#_ENREF_33) | CNN | AD vs CN (ADNI-2) | 90.7 | 93.6 | External Test | 577 | CAM | No |
|  |  | sMCI vs pMCI (ADNI-2) | 79.3 | 77.6 |  | 250 |  |  |
| Gutierrez-Becker, 2018[^34^](#_ENREF_34) | PointNet | AD vs CN | Other metrics reported |  | Hold-out Test | *1196* | Occlusion | No |
|  |  | MCI vs CN |  |  |  |  |  |  |
| He, 2021[^35^](#_ENREF_35) | CNN | AD vs CN | *[89-94]* |  | 10-fold CV |  | Occlusion | No |
| Hu, 2021[^36^](#_ENREF_36) | Autoencoder | FTD vs FTD_NC | 93.5 |  | Hold-out Test | 184 | Guided Backpropagation | No |
|  |  | AD vs AD_NC | 89.9 |  |  | 176 |  |  |
|  |  | FTD vs AD vs NC | 91.8 |  |  | 360 |  |  |
|  |  | FTD vs AD | 93.1 |  |  | 200 |  |  |
| Huang, 2021[^37^](#_ENREF_37) | CNN | AD vs CN (AIBL-1) | 88.8 | 93.4 | External Test | 386 | Backpropagation | No |
|  |  | AD vs CN (AIBL-2) | 82.7 | 97.3 |  | 197 |  |  |
|  |  | AD vs CN (OASIS) | 92.5 | 97.2 |  | 346 |  |  |
|  |  | AD vs MCI vs CN (AIBL-1) | 76.0 | 77.5 |  | 463 |  |  |
|  |  | AD vs MCI vs CN (AIBL-2) | 39.1 | 81.7 |  | 244 |  |  |
|  |  | AD vs MCI vs CN (OASIS) | 74.5 | 81.6 |  | 416 |  |  |
| Iizuka, 2019[^38^](#_ENREF_38) | CNN | DLB vs CN | 93.1 | 95.4 | Not reported |  | Grad-CAM | Yes |
|  |  | DLB vs AD | 89.3 | 93.5 |  |  |  |  |
|  |  | AD vs NL | 92.4 | 94.3 |  |  |  |  |
| Irie, 2020[^39^](#_ENREF_39) | Autoencoder | iNPH vs AD vs CN | 90 |  | LOOCV |  | Grad-CAM |  |
| Jain, 2021[^40^](#_ENREF_40) | VGG19 | ND vs MCI vs MD vs SD | 86 |  | Hold-out Test | 2000 | Grad-CAM | No |
| Jin, 2019[^41^](#_ENREF_41) | ResNet | AD vs CN | 92.0 | 94.1 | 10-fold CV |  | Attention map | Yes |
| Jin, 2020[^42^](#_ENREF_42) | ResNet | AD vs CN (ADNI) | 86.1 | 91.2 | Hold-out Test | 1116 | Attention map | Yes |
|  |  | AD vs CN (In-house) | 87.0 | 91.3 | External Test | 716 |  |  |
| Jo, 2020[^43^](#_ENREF_43) | CNN | AD vs CN | 90.8 |  | 5-fold CV |  | LRP | Yes |
| Kamal, 2021[^44^](#_ENREF_44) | Linear SVM | AD vs CN | 82.4 |  | Not reported |  | LIME | No |
| Khan, 2019[^45^](#_ENREF_45) | VGG19 | AD vs MC vs NC | 95.2 |  | 5-fold CV |  | CAM | No |
| Kroll, 2020[^46^](#_ENREF_46) | GA-NB | AD vs CN | 81.5 | 91.3 | 9-fold CV |  | Rule extraction | No |
|  |  | MCI-C vs MCI-NC | 68.8 | 74.4 |  |  |  |  |
| Lee, 2019[^47^](#_ENREF_47) | DNN | MCI vs CN | 89.2 | 95.7 | 10-fold CV |  | Activation map | No |
|  |  | sMCI vs pMCI | 88.5 | 95.7 |  |  |  |  |
| Lee, 2021[^48^](#_ENREF_48) | CNN | AD vs CN |  | 94.0 | Random Split | 70 | CAM | No |
|  |  | DWS vs CN |  | 75.0 | External Test | 110 |  |  |
| Li, 2018[^49^](#_ENREF_49) | RNN | AD vs CN |  | 86.2 | 5-fold CV |  | Attention values | No |
|  |  | MCI vs CN |  | 61.7 |  |  |  |  |
| Li, 2021[^50^](#_ENREF_50) | ResNet | AD vs CN | 95.0 | 99.5 | Hold-out Test | 60 | Weight visualisation, Grad-CAM | No |
| Lian, 2020[^51^](#_ENREF_51) | FCN | AD vs CN (ADNI-1) | 89.5 | 94.5 | Hold-out Test | 636 | CAM | Yes |
|  |  | AD vs CN (ADNI-2) | 90.3 | 95.1 | External Test | 821 |  |  |
|  |  | sMCI vs pMCI (ADNI-2) | 80.9 | 78.1 |  | 277 |  |  |
| Liu, 2019[^52^](#_ENREF_52) | VGG | AD vs CN | 86.8 | 85.7 | 5-fold CV |  | Patch-wise permutation | Yes |
| Liu, 2021 | CNN | AD vs CN | 88† |  | Hold-out Test | *83* | Counterfactual explanations | Yes |
| Mendoza-Léon, 2020[^54^](#_ENREF_54) | Autoencoder | AD vs CN | 90.0 | 92.0 | Hold-out Test | 40 | Accuracy map | No |
| Moon, 2021[^55^](#_ENREF_55) | SVM | AD vs CN | 80.1 |  | 5-fold CV |  | LIME | Yes |
|  |  | MCI vs CN | 77.1 |  |  |  |  |  |
| Morabito, 2021[^56^](#_ENREF_56) | CNN | MCI vs AD | 98.5 |  | 7-fold CV |  | Grad-CAM | No |
| Morales, 2013[^57^](#_ENREF_57) | Naïve Bayes | PDD vs PDCI | 97.0 |  | 5-fold CV |  | Selection frequency | No |
|  |  | PDD vs PDMCI | 96.6 |  |  |  |  |  |
|  |  | PDMCI vs PDCI | 90.1 |  |  |  |  |  |
|  |  | PDD vs PDMCI vs PDCI | 68.9 |  |  |  |  |  |
| Murugan, 2021[^58^](#_ENREF_58) | CNN | Mild vs Mod vs Very Mild vs No Dementia | 95.2 | 97 | Hold-out Test | *1280* | Occlusion | No |
| Nigri, 2020[^59^](#_ENREF_59) | AlexNet | AD vs CN | 92.3 |  | Hold-out Test | 575 | Swap Test, Occlusion | Yes |
| Orlenko, 2021[^60^](#_ENREF_60) | RF | AD/MCI vs CN | 62.6† |  | k-fold CV |  | Permutation, Shapley Values | Yes |
| Ortiz, 2016[^61^](#_ENREF_61) | DBN-SVM | AD vs CN | 90.0 | 95.0 | 10-fold CV |  | Weight visualisation | No |
| Ortiz-Suárez, 2017[^62^](#_ENREF_62) | CNN | AD vs CN | 82.5 | 90.0 | Bootstrapping |  | Activation map | No |
| Pohl, 2021[^63^](#_ENREF_63) | CNN | AD vs CN | 92.1 |  | Hold-out Test | 69 | LRP | Yes |
| Polsterl, 2021[^64^](#_ENREF_64) | DNN | AD vs CN | 94.2† |  | Hold-out Test | 176 | Shapley values | No |
| Qiu, 2020[^65^](#_ENREF_65) | FCN-MLP | AD vs CN (ADNI) | 96.8 | 99.6 | Hold-out Test | 83 | Accuracy map | Yes |
|  |  | AD vs CN (AIBL) | 93.2 | 97.4 | External Test | 382 |  |  |
|  |  | AD vs CN (FHS) | 79.2 | 87.6 |  | 102 |  |  |
|  |  | AD vs CN (NACC) | 85.2 | 95.4 |  | 565 |  |  |
| Ren, 2019[^66^](#_ENREF_66) | CNN | AD vs CN | 93.8 | 93.0 | 10-fold CV |  | Backpropagation | No |
|  |  | AD vs MCI | 85.3 | 80.0 |  |  |  |  |
|  |  | CN vs MCI | 88.5 | 82.0 |  |  |  |  |
| Rieke, 2018[^67^](#_ENREF_67) | CNN | AD vs CN | 77.0 | 78.0 | 5-fold CV |  | (Guided) Backpropagation, Occlusion | No |
| Ronge, 2021[^68^](#_ENREF_68) | DeepFM | AD vs MCI vs CN | 58.9† |  | 5-fold CV |  | Weight values | No |
| Ruiz, 2018[^69^](#_ENREF_69) | Greedy Algorithm | AD vs CN | 80.4 | 89.4 | 10-fold CV |  | Weight visualisation | No |
|  |  | MCI vs CN | 59.1 | 73.7 |  |  |  |  |
|  |  | AD vs MCI | 51.6 | 64.6 |  |  |  |  |
| Sarica, 2021[^70^](#_ENREF_70) | EBM | sMCI vs pMCI |  | 84.0 | Hold-out Test | 40 | LIME | No |
| Sha, 2021[^71^](#_ENREF_71) | LGP | AD vs CN | *≥80* |  | Hold-out Test | 23 | Accuracy map | No |
| Shahamat, 2020[^72^](#_ENREF_72) | CNN | AD vs CN | 85.0 |  | 5-fold CV |  | Occlusion | No |
| Shaji, 2021[^73^](#_ENREF_73) | Inception-ResNet | AD vs CN | 69.0 |  | 5-fold CV |  | Grad-CAM | No |
| Shoaip, 2021[^74^](#_ENREF_74) | Decision Trees | AD vs eMCI vs lMCI vs SMC vs CN | 92.3 |  | 10-fold CV |  | Rule extraction | No |
| Song, 2021[^75^](#_ENREF_75) | CNN | AD vs CN | 94.1 |  | 10-fold CV |  | Grad-CAM | No |
| Song, 2021[^76^](#_ENREF_76) | TSK Fuzzy | AD vs CN | 86.1 |  | Not reported |  | Rule extraction | No |
| Souillard-Mandar, 2016[^77^](#_ENREF_77) | Decision Trees | AD/aMCI vs CN |  | 93.0 | Nested 5-fold CV |  | Rule extraction | No |
|  |  | VCD vs CN |  | 88.0 |  |  |  |  |
| Sun, 2018[^78^](#_ENREF_78) | SVM+SAR | AD vs CN | 89.3 | 95.1 | Not reported |  | Weight visualisation, Selection frequency | No |
|  |  | CN vs MCI | 70.8 | 77.9 |  |  |  |  |
|  |  | AD vs MCI | 65.7 | 70.5 |  |  |  |  |
|  |  | sMCI vs pMCI | 65.4 | 68.3 |  |  |  |  |
| Thibeau-Sutre, 2020[^79^](#_ENREF_79) | CNN | AD vs CN (ADNI) | 88† |  | Hold-out Test | 200 | Occlusion | Yes |
|  |  | AD vs CN (AIBL) | 90† |  | External Test |  |  |  |
| Tian, 2021[^80^](#_ENREF_80) | SVM | AD vs CN | 82.4 |  | 5-fold CV |  | Occlusion | No |
| Vandenberghe, 2013[^81^](#_ENREF_81) | SVM | AD vs CN | Other metrics reported |  | Not reported |  | Weight visualisation | Yes |
| Velazquez, 2021[^82^](#_ENREF_82) | RF | eMCI-C vs eMCI-NC | 93.6 | 96.0 | Hold-out Test | 95 | Feature importance | No |
| Venugopalan, 2021[^83^](#_ENREF_83) | DNN-SVM | AD vs MCI vs CN | 78.0 |  | Hold-out Test | *200* | Permutation | No |
| Vigneron, 2016[^84^](#_ENREF_84) | RF | AD vs CN | 92.6 |  | Hold-out Test | 46 | Feature permutation | Yes |
|  |  | MCI vs CN | 91.8 |  |  |  |  |  |
|  |  | AD vs MCI_AD | 84.7 |  |  |  |  |  |
| Wehenkel, 2017[^85^](#_ENREF_85) | ET | sMCI vs pMCI | 71.6 | 74.0 | 10-fold CV |  | Weight visualisation, Feature importance | No |
|  |  | Dementia vs CN (OASIS) | 66.4 | 70.3 | External Test | 100 |  |  |
| Wehenkel, 2018[^86^](#_ENREF_86) | RF | sMCI vs MCI | 71.1 |  | 10-fold CV |  | Feature importance | No |
| Yang, 2018[^87^](#_ENREF_87) | ResNet | AD vs CN | 79.4 | 85.4 | 5-fold CV |  | Occlusion, Contour Map, (Grad)-CAM | No |
| Yee, 2020[^88^](#_ENREF_88) | CNN | sCN vs sDAT | 93.5 | 97.6 | 5-fold CV |  | Grad-CAM | No |
|  |  | sMCI vs pMCI | 74.7 | 81.1 | Hold-out Test | 1233 |  |  |
| Zeng, 2020[^89^](#_ENREF_89) | Decision Trees | AD vs MCI vs CN | 91.5 |  | 6-fold CV |  | Rule extraction | No |
|  |  | C vs NC | 83.9 |  |  |  |  |  |
| Zhang, 2021[^90^](#_ENREF_90) | CNN | AD vs CN (AIBL) | 95.8 | 97.9 | External Test | 467 | Attention values | No |
|  |  | AD vs CN (NACC) | 90.7 | 96.1 |  | 469 |  |  |
| Zhang, 2021[^91^](#_ENREF_91) | ResNet | AD vs CN | 91.3 | 98.4 | 5-fold CV | 258 | Grad-CAM | No |
|  |  | sMCI vs pMCI | 82.1 | 92.0 |  |  |  |  |
|  |  | AD vs CN (ADNI-2) | 90.9 | 95.2 | External Test | 258 |  |  |
|  |  | AD vs CN (ADNI-3) | 89.2 | 88.9 |  |  |  |  |
| Zhu, 2022[^92^](#_ENREF_92) | GCN | AD vs CN | *[90-95]* |  | Repeated Random Split |  | Selection frequency | Yes |

**References**

1. Abrol A, Bhattarai M, Fedorov A, Du Y, Plis S, Calhoun V. Deep residual learning for neuroimaging: An application to predict progression to Alzheimer's disease. Article. *Journal of Neuroscience Methods*. 2020;339108701. doi:10.1016/j.jneumeth.2020.108701

2. Abuhmed T, El-Sappagh S, Alonso JM. Robust hybrid deep learning models for Alzheimer's progression detection. *Knowledge-Based Systems*. Feb 2021;213106688. doi:10.1016/j.knosys.2020.106688

3. Achilleos KG, Leandrou S, Prentzas N, Kyriacou PA, Kakas AC, Pattichis CS. Extracting Explainable Assessments of Alzheimer’s disease via Machine Learning on brain MRI imaging data. 2020:1036-1041.

4. Adel T, Cohen T, Caan M, Welling M, Grp AGS, Alzheimer's Dis N. 3D scattering transforms for disease classification in neuroimaging. *Neuroimage-Clinical*. 2017;14:506-517. doi:10.1016/j.nicl.2017.02.004

5. Azcona E, Besson P, Wu Y, et al. Interpretation of Brain Morphology in Association to Alzheimer's Disease Dementia Classification Using Graph Convolutional Networks on Triangulated Meshes. *Shape Med Imaging (2020)*. Oct 2020;12474:95-107. doi:10.1007/978-3-030-61056-2_8

6. Bae J, Stocks J, Heywood A, et al. Transfer learning for predicting conversion from mild cognitive impairment to dementia of Alzheimer's type based on a three-dimensional convolutional neural network. *Neurobiol Aging*. Mar 2021;99:53-64. doi:10.1016/j.neurobiolaging.2020.12.005

7. Balagopalan A, Eyre B, Robin J, Rudzicz F, Novikova J. Comparing Pre-trained and Feature-Based Models for Prediction of Alzheimer's Disease Based on Speech. *Frontiers in Aging Neuroscience*. Apr 2021;13635945. doi:10.3389/fnagi.2021.635945

8. Bang S, Son S, Roh H, et al. Quad-phased data mining modeling for dementia diagnosis. *Bmc Medical Informatics and Decision Making*. May 2017;1760. doi:10.1186/s12911-017-0451-3

9. Beebe-Wang N, Okeson A, Althoff T, Lee SI. Efficient and Explainable Risk Assessments for Imminent Dementia in an Aging Cohort Study. *IEEE Journal of Biomedical and Health Informatics*. 2021/7// 2021;25(7):2409-2420. doi:10.1109/JBHI.2021.3059563

10. Ben Miled Z, Haas K, Black CM, et al. Predicting dementia with routine care EMR data. *Artificial intelligence in medicine*. 2020/1// 2020;102:101771-101771. doi:10.1016/j.artmed.2019.101771

11. Bloch L, Friedrich CM. Developing a Machine LearningWorkflow to Explain Black-box Models for Alzheimer's Disease Classification. 2021:87-99.

12. Bloch L, Friedrich CM. Data analysis with Shapley values for automatic subject selection in Alzheimer’s disease data sets using interpretable machine learning. *Alzheimer's Research and Therapy*. 2021/12// 2021;13(1)doi:10.1186/s13195-021-00879-4

13. Böhle M, Eitel F, Weygandt M, Ritter K. Layer-wise relevance propagation for explaining deep neural network decisions in MRI-based Alzheimer's disease classification. *Frontiers in Aging Neuroscience*. 2019;10(JUL)doi:10.3389/fnagi.2019.00194

14. Choi H, Kim YK, Yoon EJ, Lee JY, Lee DS. Cognitive signature of brain FDG PET based on deep learning: domain transfer from Alzheimer's disease to Parkinson's disease. *European Journal of Nuclear Medicine and Molecular Imaging*. Feb 2020;47(2):403-412. doi:10.1007/s00259-019-04538-7

15. Chyzhyk DS, A.: Graña, M. Evolutionary ELM wrapper feature selection for Alzheimer's disease CAD on anatomical brain MRI. Article. *Neurocomputing*. 2014;128:73-80. doi:10.1016/j.neucom.2013.01.065

16. Cuingnet R, Glaunès JA, Chupin M, Benali H, Colliot O. Spatial and Anatomical Regularization of SVM: A General Framework for Neuroimaging Data. *IEEE Trans Pattern Anal Mach Intell*. Mar 2013;35(3):682-96. doi:10.1109/tpami.2012.142

17. Danso SO, Zeng Z, Muniz-Terrera G, Ritchie CW. Developing an Explainable Machine Learning-Based Personalised Dementia Risk Prediction Model: A Transfer Learning Approach With Ensemble Learning Algorithms. *Frontiers in big data*. 2021/5// 2021;4:613047-613047. doi:10.3389/fdata.2021.613047

18. Das D, Ito J, Kadowaki T, Tsuda K. An interpretable machine learning model for diagnosis of Alzheimer's disease. *Peerj*. Mar 2019;7e6543. doi:10.7717/peerj.6543

19. Ding Y, Sohn JH, Kawczynski MG, et al. A Deep Learning Model to Predict a Diagnosis of Alzheimer Disease by Using (18)F-FDG PET of the Brain. *Radiology*. Feb 2019;290(2):456-464. doi:10.1148/radiol.2018180958

20. Ding Z, Liu Y, Tian X, et al. Multi-resolution 3D-HOG feature learning method for Alzheimer's Disease diagnosis. Article. *Computer Methods and Programs in Biomedicine*. 2022;214106574. doi:10.1016/j.cmpb.2021.106574

21. Doborjeh MD, Z.: Merkin, A.: Bahrami, H.: Sumich, A.: Krishnamurthi, R.: Medvedev, O. N.: Crook-Rumsey, M.: Morgan, C.: Kirk, I.: Sachdev, P. S.: Brodaty, H.: Kang, K.: Wen, W.: Feigin, V.: Kasabov, N. Personalised predictive modelling with brain-inspired spiking neural networks of longitudinal MRI neuroimaging data and the case study of dementia. *Neural Networks*. Dec 2021;144:522-539. doi:10.1016/j.neunet.2021.09.013

22. Donini M, Monteiro JM, Pontil M, et al. Combining heterogeneous data sources for neuroimaging based diagnosis: re-weighting and selecting what is important. Article. *NeuroImage*. 2019;195:215-231. doi:10.1016/j.neuroimage.2019.01.053

23. Durongbhan PZ, Y. F.: Chen, L. Y.: Zis, P.: De Marco, M.: Unwin, Z. C.: Venneri, A.: He, X. X.: Li, S.: Zhao, Y. T.: Blackburn, D. J.: Sarrigiannis, P. G. A Dementia Classification Framework Using Frequency and Time-Frequency Features Based on EEG Signals. *Ieee Transactions on Neural Systems and Rehabilitation Engineering*. May 2019;27(5):826-835. doi:10.1109/tnsre.2019.2909100

24. Dyrba M, Pallath AH, Marzban EN. Comparison of CNN visualization methods to aid model interpretability for detecting alzheimer’s disease. In: *Informatik aktuell*. 2020:307-312.

25. Dyrba M, Hanzig M, Altenstein S, et al. Improving 3D convolutional neural network comprehensibility via interactive visualization of relevance maps: evaluation in Alzheimer's disease. *Alzheimers Research & Therapy*. Nov 2021;13(1)191. doi:10.1186/s13195-021-00924-2

26. Eitel F, Ritter K, Alzheimer's Dis N. Testing the Robustness of Attribution Methods for Convolutional Neural Networks in MRI-Based Alzheimer's Disease Classification. 2019:3-11.

27. El-Sappagh S, Alonso JM, Islam SMR, Sultan AM, Kwak KS. A multilayer multimodal detection and prediction model based on explainable artificial intelligence for Alzheimer’s disease. *Scientific Reports*. 2021/12// 2021;11(1)doi:10.1038/s41598-021-82098-3

28. Esmaeilzadeh S, Belivanis DI, Pohl KM, Adeli E. End-To-End Alzheimer's Disease Diagnosis and Biomarker Identification. 2018:337-345.

29. Etminani K, Soliman A, Davidsson A, et al. A 3D deep learning model to predict the diagnosis of dementia with Lewy bodies, Alzheimer's disease, and mild cognitive impairment using brain 18F-FDG PET. *Eur J Nucl Med Mol Imaging*. Jul 30 2021;doi:10.1007/s00259-021-05483-0

30. Fan Z, Li J, Zhang L, et al. U-net based analysis of MRI for Alzheimer’s disease diagnosis. Article. *Neural Computing and Applications*. 2021;33(20):13587-13599. doi:10.1007/s00521-021-05983-y

31. Ford E, Starlinger J, Rooney P, et al. Could dementia be detected from UK primary care patients' records by simple automated methods earlier than by the treating physician? A retrospective case-control study. Article. *Wellcome Open Research*. 2020;5120. doi:10.12688/wellcomeopenres.15903.1

32. Gu P, Xu X, Luo Y, Wang P, Lu J. BCN-GCN: A Novel Brain Connectivity Network Classification Method via Graph Convolution Neural Network for Alzheimer’s Disease. Springer International Publishing; 2021:657-668.

33. Guan H, Wang C, Cheng J, Jing J, Liu T. A parallel attention-augmented bilinear network for early magnetic resonance imaging-based diagnosis of Alzheimer's disease. Article. *Human Brain Mapping*. 2022;43(2):760-772. doi:10.1002/hbm.25685

34. Gutierrez-Becker B, Wachinger C. Deep Multi-structural Shape Analysis: Application to Neuroanatomy. 2018:523-531.

35. He YX, Wu J, Zhou L, Chen Y, Li F, Qian HJ. Quantification of Cognitive Function in Alzheimer's Disease Based on Deep Learning. *Frontiers in Neuroscience*. Mar 2021;15651920. doi:10.3389/fnins.2021.651920

36. Hu J, Qing Z, Liu R, et al. Deep Learning-Based Classification and Voxel-Based Visualization of Frontotemporal Dementia and Alzheimer’s Disease. Article. *Frontiers in Neuroscience*. 2021;14626154. doi:10.3389/fnins.2020.626154

37. Huang YL, Yang HC, Lee CC. Federated Learning via Conditional Mutual Learning for Alzheimer's Disease Classification on T1w MRI. *Annu Int Conf IEEE Eng Med Biol Soc*. Nov 2021;2021:2427-2432. doi:10.1109/embc46164.2021.9630382

38. Iizuka T, Fukasawa M, Kameyama M. Deep-learning-based imaging-classification identified cingulate island sign in dementia with Lewy bodies. *Scientific Reports*. Jun 2019;98944. doi:10.1038/s41598-019-45415-5

39. Irie R, Otsuka Y, Hagiwara A, et al. A Novel Deep Learning Approach with a 3D Convolutional Ladder Network for Differential Diagnosis of Idiopathic Normal Pressure Hydrocephalus and Alzheimer's Disease. *Magnetic Resonance in Medical Sciences*. 2020;19(4):351-358. doi:10.2463/mrms.mp.2019-0106

40. Jain V, Nankar O, Jerrish DJ, Gite S, Patil S, Kotecha K. A Novel AI-Based System for Detection and Severity Prediction of Dementia Using MRI. *Ieee Access*. 2021;9:154324-154346. doi:10.1109/access.2021.3127394

41. Jin D, Xu J, Zhao K, et al. Attention-based 3D Convolutional Network for Alzheimer's Disease Diagnosis and Biomarkers Exploration. 2019:1047-1051.

42. Jin D, Zhou B, Han Y, et al. Generalizable, Reproducible, and Neuroscientifically Interpretable Imaging Biomarkers for Alzheimer's Disease. Article. *Advanced Science*. 2020;7(14)2000675. doi:10.1002/advs.202000675

43. Jo T, Nho K, Risacher SL, Saykin AJ. Deep learning detection of informative features in tau PET for Alzheimer's disease classification. *BMC Bioinformatics*. Dec 28 2020;21(Suppl 21):496. doi:10.1186/s12859-020-03848-0

44. Kamal MS, Northcote A, Chowdhury L, Dey N, Crespo RG, Herrera-Viedma E. Alzheimer's Patient Analysis Using Image and Gene Expression Data and Explainable-AI to Present Associated Genes. *Ieee Transactions on Instrumentation and Measurement*. 2021;702513107. doi:10.1109/tim.2021.3107056

45. Khan NM, Abraham N, Hon M. Transfer Learning With Intelligent Training Data Selection for Prediction of Alzheimer's Disease. *Ieee Access*. 2019;7:72726-72735. doi:10.1109/access.2019.2920448

46. Kroll JP, Eickhoff SB, Hoffstaedter F, Patil KR. Evolving complex yet interpretable representations: Application to Alzheimer's diagnosis and prognosis. In: *2020 IEEE Congress on Evolutionary Computation, CEC 2020 - Conference Proceedings*. 2020:

47. Lee E, Choi JS, Kim M, Suk HI. Toward an interpretable Alzheimer's disease diagnostic model with regional abnormality representation via deep learning. Article. *NeuroImage*. 2019;202116113. doi:10.1016/j.neuroimage.2019.116113

48. Lee R, Choi H, Park KY, Kim JM, Seok JW. Prediction of post-stroke cognitive impairment using brain FDG PET: deep learning-based approach. *Eur J Nucl Med Mol Imaging*. Oct 2 2021;doi:10.1007/s00259-021-05556-0

49. Li J, Rong Y, Meng HL, et al. TATC Predicting Alzheimer's Disease with Actigraphy Data. 2018:509-518.

50. Li Q, Yang MQ. Comparison of machine learning approaches for enhancing Alzheimer's disease classification. *Peerj*. Feb 2021;9e10549. doi:10.7717/peerj.10549

51. Lian CL, M.: Zhang, J.: Shen, D. Hierarchical fully convolutional network for joint atrophy localization and Alzheimer's disease diagnosis using structural MRI. Article. *IEEE Transactions on Pattern Analysis and Machine Intelligence*. 2020;42(4):880-893. 8585141. doi:10.1109/TPAMI.2018.2889096

52. Liu Y, Li Z, Ge Q, Lin N, Xiong M. Deep Feature Selection and Causal Analysis of Alzheimer’s Disease. Article. *Frontiers in Neuroscience*. 2019;131198. doi:10.3389/fnins.2019.01198

53. Liu Z, Adeli E, Pohl KM, Zhao Q. Going Beyond Saliency Maps: Training Deep Models to Interpret Deep Models. Springer International Publishing; 2021:71-82.

54. Mendoza-Léon R, Puentes J, Uriza LF, Hernández Hoyos M. Single-slice Alzheimer's disease classification and disease regional analysis with Supervised Switching Autoencoders. Article. *Computers in Biology and Medicine*. 2020;116103527. doi:10.1016/j.compbiomed.2019.103527

55. Moon S, Lee H. JDSNMF: Joint Deep Semi-Non-Negative Matrix Factorization for Learning Integrative Representation of Molecular Signals in Alzheimer's Disease. *Journal of Personalized Medicine*. Aug 2021;11(8)686. doi:10.3390/jpm11080686

56. Morabito FC, Ieracitano C, Mammone N. An explainable Artificial Intelligence approach to study MCI to AD conversion via HD-EEG processing. Article. *Clinical EEG and Neuroscience*. 2021;doi:10.1177/15500594211063662

57. Morales DA, Vives-Gilabert Y, Gómez-Ansón B, et al. Predicting dementia development in Parkinson's disease using Bayesian network classifiers. Article. *Psychiatry Research - Neuroimaging*. 2013;213(2):92-98. doi:10.1016/j.pscychresns.2012.06.001

58. Murugan S, Venkatesan C, Sumithra MG, et al. DEMNET: A Deep Learning Model for Early Diagnosis of Alzheimer Diseases and Dementia From MR Images. *Ieee Access*. 2021;9:90319-90329. doi:10.1109/access.2021.3090474

59. Nigri E, Ziviani N, Cappabianco F, Antunes A, Veloso A, Ieee. Explainable Deep CNNs for MRI-Based Diagnosis of Alzheimer's Disease. 2020:

60. Orlenko AM, J. H. A comparison of methods for interpreting random forest models of genetic association in the presence of non-additive interactions. *Biodata Mining*. Jan 2021;14(1)9. doi:10.1186/s13040-021-00243-0

61. Ortiz AM, J.: Górriz, J. M.: Ramírez, J. Ensembles of Deep Learning Architectures for the Early Diagnosis of the Alzheimer's Disease. Conference Paper. *International Journal of Neural Systems*. 2016;26(7)1650025. doi:10.1142/S0129065716500258

62. Ortiz-Suárez JM, Ramos-Pollán R, Romero E. Exploring Alzheimer's anatomical patterns through convolutional networks. In: *Proceedings of SPIE - The International Society for Optical Engineering*. 2017:

63. Pohl T, Jakab M, Benesova W. Interpretability of deep neural networks used for the diagnosis of Alzheimer's disease. Article. *International Journal of Imaging Systems and Technology*. 2021;doi:10.1002/ima.22657

64. Polsterl S, Aigner C, Wachinger C. Scalable, Axiomatic Explanations of Deep Alzheimer's Diagnosis from Heterogeneous Data. 2021:434-444.

65. Qiu S, Joshi PS, Miller MI, et al. Development and validation of an interpretable deep learning framework for Alzheimer's disease classification. *Brain*. 2020/6// 2020;143(6):1920-1933. doi:10.1093/brain/awaa137

66. Ren FJ, Yang CH, Qiu Q, et al. Exploiting Discriminative Regions of Brain Slices Based on 2D CNNs for Alzheimer's Disease Classification. *Ieee Access*. 2019;7:181423-181433. doi:10.1109/access.2019.2920241

67. Rieke J, Eitel F, Weygandt M, Haynes J-D, Ritter K. Visualizing Convolutional Networks for MRI-Based Diagnosis of Alzheimer’s Disease. Springer International Publishing; 2018:24-31.

68. Ronge R, Nho K, Wachinger C, Pölsterl S. Alzheimer’s Disease Diagnosis via Deep Factorization Machine Models. Springer International Publishing; 2021:624-633.

69. Ruiz ER, J.: Górriz, J. M.: Casillas, J. Alzheimer's disease computer-aided diagnosis: Histogram-based analysis of regional mri volumes for feature selection and classification. Article. *Journal of Alzheimer's Disease*. 2018;65(3):819-842. doi:10.3233/JAD-170514

70. Sarica A, Quattrone A, Quattrone A. Explainable Boosting Machine for Predicting Alzheimer’s Disease from MRI Hippocampal Subfields. Springer International Publishing; 2021:341-350.

71. Sha C, Cuperlovic-Culf M, Hu T. SMILE: systems metabolomics using interpretable learning and evolution. *BMC Bioinformatics*. 2021/12// 2021;22(1)doi:10.1186/s12859-021-04209-1

72. Shahamat H, Saniee Abadeh M. Brain MRI analysis using a deep learning based evolutionary approach. Article. *Neural Networks*. 2020;126:218-234. doi:10.1016/j.neunet.2020.03.017

73. Shaji S, Ganapathy N, Swaminathan R. Classification of Alzheimer Condition using MR Brain Images and Inception-Residual Network Model. Article. *Current Directions in Biomedical Engineering*. 2021;7(2):763-766. doi:10.1515/cdbme-2021-2195

74. Shoaip N, Rezk A, El-Sappagh S, Abuhmed T, Barakat S, Elmogy M. Alzheimer's Disease Diagnosis Based on a Semantic Rule-Based Modeling and Reasoning Approach. *Cmc-Computers Materials & Continua*. 2021;69(3):3531-3548. doi:10.32604/cmc.2021.019069

75. Song J, Zheng J, Li P, Lu X, Zhu G, Shen P. An Effective Multimodal Image Fusion Method Using MRI and PET for Alzheimer's Disease Diagnosis. *Front Digit Health*. 2021;3:637386. doi:10.3389/fdgth.2021.637386

76. Song XJ, Gu F, Wang XD, Ma SH, Wang L. Interpretable Recognition for Dementia Using Brain Images. *Frontiers in Neuroscience*. Sep 2021;15748689. doi:10.3389/fnins.2021.748689

77. Souillard-Mandar W, Davis R, Rudin C, et al. Learning classification models of cognitive conditions from subtle behaviors in the digital Clock Drawing Test. *Machine Learning*. Mar 2016;102(3):393-441. doi:10.1007/s10994-015-5529-5

78. Sun Z, Qiao YC, Lelieveldt BPF, Staring M, Alzheimers Dis NeuroImaging I. Integrating spatial-anatomical regularization and structure sparsity into SVM: Improving interpretation of Alzheimer's disease classification. *Neuroimage*. Sep 2018;178:445-460. doi:10.1016/j.neuroimage.2018.05.051

79. Thibeau-Sutre E, Colliot O, Dormont D, Burgos N. Visualization approach to assess the robustness of neural networks for medical image classification. In: *Progress in Biomedical Optics and Imaging - Proceedings of SPIE*. 2020:

80. Tian J, Smith G, Guo H, et al. Modular machine learning for Alzheimer's disease classification from retinal vasculature. Article. *Scientific Reports*. 2021;11(1)238. doi:10.1038/s41598-020-80312-2

81. Vandenberghe R, Nelissen N, Salmon E, et al. Binary classification of 18F-flutemetamol PET using machine learning: Comparison with visual reads and structural MRI. Article. *NeuroImage*. 2013;64(1):517-525. doi:10.1016/j.neuroimage.2012.09.015

82. Velazquez M, Lee Y, for the Alzheimer's Disease Neuroimaging I. Random forest model for feature-based Alzheimer’s disease conversion prediction from early mild cognitive impairment subjects. Article. *PLoS ONE*. 2021;16(4 April)e0244773. doi:10.1371/journal.pone.0244773

83. Venugopalan J, Tong L, Hassanzadeh HR, Wang MD. Multimodal deep learning models for early detection of Alzheimer's disease stage. *Scientific Reports*. Feb 2021;11(1)3254. doi:10.1038/s41598-020-74399-w

84. Vigneron V, Kodewitz A, Tome AM, Lelandais S, Lang E. Alzheimer's Disease Brain Areas: The Machine Learning Support for Blind Localization. *Current Alzheimer Research*. 2016;13(5):498-508. doi:10.2174/1567205013666160314144822

85. Wehenkel M, Bastin C, Phillips C, Geurts P, Ieee. Tree Ensemble Methods and Parcelling to Identify Brain Areas Related to Alzheimer's Disease. 2017:

86. Wehenkel M, Sutera A, Bastin C, Geurts P, Phillips C. Random Forests Based Group Importance Scores and Their Statistical Interpretation: Application for Alzheimer's Disease. *Frontiers in Neuroscience*. Jun 2018;12411. doi:10.3389/fnins.2018.00411

87. Yang C, Rangarajan A, Ranka S. Visual Explanations From Deep 3D Convolutional Neural Networks for Alzheimer's Disease Classification. *AMIA Annu Symp Proc*. 2018;2018:1571-1580.

88. Yee E, Popuri K, Beg MF. Quantifying brain metabolism from FDG-PET images into a probability of Alzheimer's dementia score. *Hum Brain Mapp*. Jan 2020;41(1):5-16. doi:10.1002/hbm.24783

89. Zeng Z, Shen Z, Hsiang BTT, et al. Explainable and argumentation-based decision making with qualitative preferences for diagnostics and prognostics of alzheimer's disease. In: *17th International Conference on Principles of Knowledge Representation and Reasoning, KR 2020*. 2020:814-824.

90. Zhang Q, Du Q, Liu GH. A whole-process interpretable and multi-modal deep reinforcement learning for diagnosis and analysis of Alzheimer's disease *. *Journal of Neural Engineering*. Dec 2021;18(6)066032. doi:10.1088/1741-2552/ac37cc

91. Zhang X, Han L, Zhu W, Sun L, Zhang D. An Explainable 3D Residual Self-Attention Deep Neural Network For Joint Atrophy Localization and Alzheimer's Disease Diagnosis using Structural MRI. Article. *IEEE Journal of Biomedical and Health Informatics*. 2021;doi:10.1109/JBHI.2021.3066832

92. Zhu Y, Ma J, Yuan C, Zhu X. Interpretable learning based Dynamic Graph Convolutional Networks for Alzheimer's Disease analysis. Article. *Information Fusion*. 2022;77:53-61. doi:10.1016/j.inffus.2021.07.013
